# Endothelial Sp1/Sp3 are essential to the effect of captopril on blood pressure in male mice

**DOI:** 10.1038/s41467-023-41567-1

**Published:** 2023-09-21

**Authors:** Hanlin Lu, Xiuxin Jiang, Lifan He, Xuyang Ji, Xinyun Li, Shaozhuang Liu, Yuanyuan Sun, Xiaoteng Qin, Xiwen Xiong, Sjaak Philipsen, Bo Xi, Meng Zhang, Jianmin Yang, Cheng Zhang, Yun Zhang, Wencheng Zhang

**Affiliations:** 1 National Key Laboratory for Innovation and Transformation of Luobing Theory; The Key Laboratory of Cardiovascular Remodeling and Function Research, Chinese Ministry of Education, Chinese National Health Commission and Chinese Academy of Medical Sciences; Department of Cardiology, Qilu Hospital of Shandong University, Jinan, China; 2 Department of Bariatric and Metabolic Surgery, General Surgery, Qilu Hospital, Cheeloo College of Medicine, Shandong University, Jinan, China; 3 School of Forensic Medicine, Xinxiang Medical University, Xinxiang, Henan China; 4 Department of Cell Biology, Erasmus MC, Rotterdam, The Netherlands; 5 Department of Epidemiology, School of Public Health, Cheeloo College of Medicine, Shandong University, Jinan, China

**Keywords:** Hypertension, Vascular diseases

## Abstract

Endothelial dysfunction represents a major cardiovascular risk factor for hypertension. Sp1 and Sp3 belong to the specificity protein and Krüppel-like transcription factor families. They are ubiquitously expressed and closely associated with cardiovascular development. We investigate the role of Sp1 and Sp3 in endothelial cells in vivo and evaluate whether captopril, an angiotensin-converting enzyme inhibitor (ACEI), targets Sp1/Sp3 to exert its effects. Inducible endothelial-specific Sp1/Sp3 knockout mice are generated to elucidate their role in endothelial cells. Tamoxifen-induced deletion of endothelial Sp1 and Sp3 in male mice decreases the serum nitrite/nitrate level, impairs endothelium-dependent vasodilation, and causes hypertension and cardiac remodeling. The beneficial actions of captopril are abolished by endothelial-specific deletion of Sp1/Sp3, indicating that they may be targets for ACEIs. Captopril increases Sp1/Sp3 protein levels by recruiting histone deacetylase 1, which elevates deacetylation and suppressed degradation of Sp1/Sp3. Sp1/Sp3 represents innovative therapeutic target for captopril to prevent cardiovascular diseases.

## Abstract (web summary)

Hypertension is the leading risk factor for cardiovascular disease, which represents the top cause of morbidity and mortality worldwide. Here, the authors show deletion of endothelial Sp1 and Sp3 leads to a disruption in endothelium-dependent vasodilation and the onset of hypertension, which abolishes the beneficial actions of captopril.

## Introduction

Hypertension is the leading risk factor for cardiovascular disease, which represents the top cause of morbidity and mortality worldwide^1,2^. Despite extensive recent studies discussing the multifactorial nature of hypertension, it is important to further explore its pathogenesis and search for new therapeutic targets^3–6^. Among the numerous etiologies of hypertension, endothelial dysfunction is a common cause and is considered a precursor to the development of micro- and macro-vascular complications^7^. The endothelium is a selective permeable barrier between the bloodstream and the outer vascular wall, but also functions as a critical homeostatic organ, fundamental for regulating vascular tone and blood pressure^8^. AMP-activated protein kinase (AMPK) plays an important role in various physiological and pathological processes. AMPK downregulates the expression of caveolin-1 and increases endothelial nitric oxide synthase (eNOS) activity^9^. However, the mechanisms involved in regulating AMPK expression remain to be explored.

Epigenomics is one of the latest approaches used in hypertension research^6^. Sp1 and Sp3 are members of the specificity protein/Krüppel-like factor (Sp/KLF) transcription factor family, which comprises proteins with three zinc fingers and recognizes G-rich promoter elements (GC-box and the related GT-box). Sp1 and Sp3 are ubiquitously expressed in mammalian cell types^10–12^. Sp1-knockout mice did not survive beyond embryonic day 10.5 and showed a broad range of abnormalities^11^. Sp3-knockout mice were growth retarded, which resulted in death prenatally or at birth due to complications including cardiac malformations^12,13^. In vivo, deletion of the Sp genes revealed distinct functions of Sp1 and Sp3; however, they have also shown overlapping functions in the early developmental stages in the hematopoietic system^13,14^. Endothelial Sp1/Sp3 regulated angiogenesis in retinal, pathological, and tumor models^15^. Considering the significance of Sp1/Sp3 in cardiovascular development and angiogenesis, their role in endothelial cells and related vascular diseases require further investigation.

The endothelium is increasingly becoming a surrogate endpoint of the therapeutic approach against cardiovascular risk factors, as demonstrated by its inclusion among markers of organ damage^16^. Several cardiovascular drugs, including angiotensin-converting enzyme inhibitor (ACEI), ameliorate endothelial dysfunction by their supposed pleiotropic and ancillary properties^17,18^. ACEIs are important therapeutic agents widely used for treating cardiovascular and renal diseases, with proven effects in patients worldwide^19–22^. ACEIs inhibit Ang II release and bradykinin inactivation; however, the clinical benefits of these actions have not been completely elucidated. Apart from their effects on ACE, ACEIs have a direct effect on the bradykinin B1 receptor to elevate NO release from endothelial cells^23^, although the detailed mechanism is unclear. We have recently reported the proangiogenic effect of ACEI was abolished in Sp1/Sp3-deletion mice^15^. Here, we aimed to elucidate whether captopril, a clinical ACEI, exerts its antihypertensive effects by targeting endothelial Sp1 and Sp3.

Here, we show that targeted deletion of endothelial Sp1 and Sp3 in male mice leads to a reduction in serum nitrite/nitrate levels, a disruption in endothelium-dependent vasodilation, and the onset of hypertension and cardiac remodeling. Furthermore, the favorable effects of captopril are negated by the endothelial deletion of Sp1/Sp3.

## Results

### Sp1 and Sp3 are reduced in the arteries of hypertensive patients and angiotensin II (Ang II)–induced hypertensive mice

**Fig. 1:**
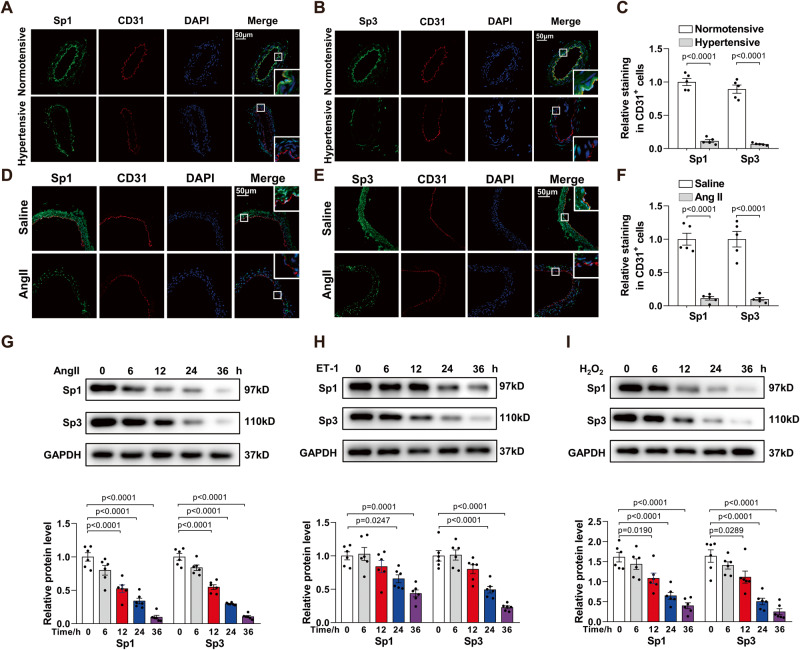
Sp1 and Sp3 levels were reduced in arteries of hypertensive patients and angiotensin II (Ang II)-induced hypertensive mice. **A**, **B** Representative immunofluorescent (IF) staining of **A** Sp1 and **B** Sp3 in mesenteric arteries from hypertensive and healthy individuals. CD31 is an endothelial cell marker. Scale bar: 50 μm. **C** Quantitative data analysis of (**A**) and (**B**). *n* = 5. **D**, **E** Representative IF staining of **D** Sp1 and **E** Sp3 in aortas from mice treated with saline or Ang II. CD31 is an endothelial cell marker. Scale bar: 50 μm. **F** Quantitative data analysis of (**D**) and (**E**). *n* = 5. Western blot (WB) analysis of Sp1 and Sp3 in HUVECs treated with **G** Ang II (10^−6 ^mol/L, 0–36 h); **H** ET-1 (10^−7 ^mol/L, 0–36 h) and **I** H_2_O_2_ (10^−4 ^mol/L, 0–36 h). *n* = 6. Data are presented as mean ± SEM. Two-tailed Student unpaired *t* test for **C** and **F**. One-way ANOVA followed by Bonferroni’s post hoc test for (**G**) to (**I**).

To determine whether Sp1 and Sp3 are involved in the pathogenesis of endothelial dysfunction and hypertension, we detected their expression in the endothelium of mesenteric arteries from hypertensive patients and hypertensive mouse arteries. Immunofluorescence (IF) staining revealed that Sp1 and Sp3 were mainly expressed in the endothelial nucleus upon colocalization of Sp1/Sp3 with DAPI (Fig. 1A–F). In the endothelium of mesenteric arteries from patients with hypertension, Sp1 and Sp3 were notably downregulated when compared to those from healthy individuals (Fig. 1A–C). The levels of Sp1 and Sp3 also decreased in the aortic endothelial cells of Ang II-induced hypertensive mice (Fig. 1D–F). mRNA levels of Sp1 and Sp3 were not influenced by Ang II in isolated mouse aortic endothelial cells (MAECs) (Fig. S1A). Thus, Sp1/Sp3 may be involved in the pathogenesis of hypertension. Given the critical roles of Ang II, endothelin-1 (ET-1), and H_2_O_2_ in hypertension and vascular diseases, we aimed to explore whether they regulate the expression of Sp1 and Sp3 in endothelial cells. In cultured human umbilical vein endothelial cells (HUVECs), Ang II, ET-1, or H_2_O_2_ markedly decreased Sp1 and Sp3 expression (Fig. 1G–I). Therefore, the reduction of Sp1 and Sp3 expression may crucially contribute to endothelial dysfunction and hypertension.

### Sp1/Sp3 depletion in EC aggravates hypertension and endothelial dysfunction in mice

**Fig. 2:**
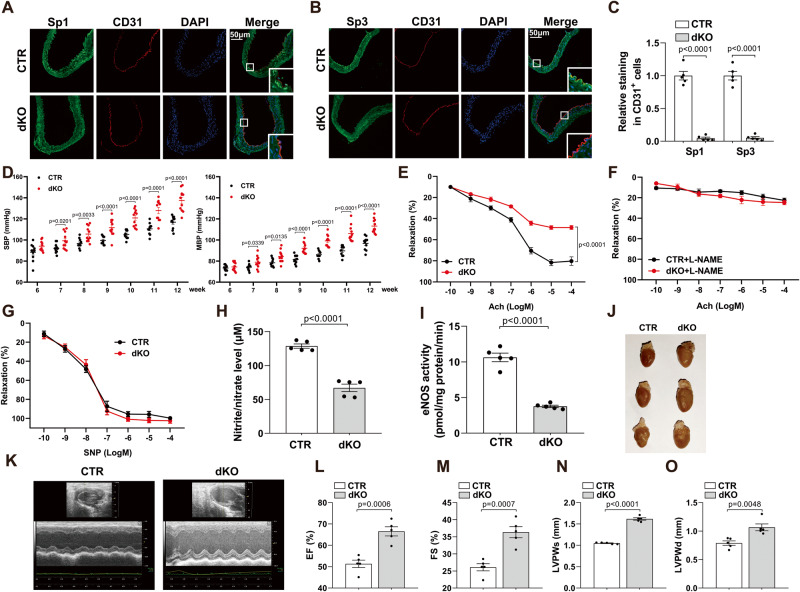
Generation and analysis of endothelial-specific Sp1/Sp3 deletion mice. **A**, **B** Representative IF staining of **A** Sp1 and **B** Sp3 in aortas from CTR and dKO mice. CD31 is used as an endothelial cell marker. Scale bar: 50 μm. **C** Quantitative data analysis of (**A**) and (**B**). *n* = 5. **D** Systolic (SBP) and mean blood pressure (MBP) in CTR and dKO mice. *n* = 10–11. **E**–**G** Vascular reactivity of mesenteric arteries from CTR and dKO mice to acetylcholine (Ach) with or without (L-NAME, 10^−4 ^mol/L, 30 min) pretreatment and sodium nitroprusside (SNP). *n* = 5. **H** Serum nitrite/nitrate level from CTR and dKO mice. *n* = 5. **I** eNOS activity in mouse lung endothelial cells (MLECs) from CTR and dKO mice. *n* = 5. **J** Representative hearts from CTR and dKO mice. **K**–**O** Representative echographic images and quantification obtained from M-mode from CTR and dKO mice. EF, ejection fraction; FS, fraction shortening; LVPWd, left ventricular posterior wall; LVPWs, systolic left ventricular posterior wall. *n* = 5. Data are presented as mean ± SEM. Two-way ANOVA followed by Bonferroni post hoc test for (**E**) to (**G**). Two-tailed Student unpaired *t* test for (**C**), (**D**), (**H**), (**I**), (**L**), (**M**), (**N**), and (**O**).

To elucidate the biological significance of Sp1 and Sp3 in vascular endothelial cells in vivo, *Sp1*^*fl/fl*^*/Sp3*^*fl/fl*^ mice were crossbred with *VE-CAD-CreER*^*T2*^ mice to generate *VE-CAD-CreER*^*T2+*^*/Sp1*^*fl/fl*^*/Sp3*^*fl/fl*^ mice, which were intraperitoneally injected with tamoxifen (1 mg/day) for 5 consecutive days to generate Sp1/Sp3^ECKO^ mice (dKO mice). Littermate *VE-CAD-CreER*^*T2-*^*/Sp1*^*fl/fl*^*/Sp3*^*fl/fl*^ mice were treated with the same dose of tamoxifen as controls (CTR mice). We examined the knockout efficiency of Sp1/Sp3 in vascular endothelial cells. IF assays revealed a significant reduction in Sp1/Sp3 content in the aortic endothelia of dKO versus CTR mice (Fig. 2A–C), which confirms the effective deletion of Sp1/Sp3 in dKO mice.

To characterize the phenotype of dKO mice, blood pressure was measured: dKO mice showed a distinct increase in systolic blood pressure (SBP) and mean blood pressure (MBP) at 7–12 weeks of age (Fig. 2D). Endothelial function was evaluated via acetylcholine (Ach)-induced relaxation in mesenteric arteries. The vasorelaxation to Ach at 10^−10^ to 10^–4^ mol/L was significantly lower in mesenteric arteries from dKO versus CTR mice (Fig. 2E). Ach elicited similar responses between the mice when mesenteric arteries were pretreated with L-nitro-arginine methyl ester (L-NAME, 10^–4^ mol/L), a nonselective NO synthase inhibitor, for 30 min (Fig. 2F). Mesenteric arteries showed no difference in relaxation caused by sodium nitroprusside (SNP, 10^−10^ to 10^–4^ mol/L), an endothelium-independent agent (Fig. 2G). Serum nitrite/nitrate level was decreased by approximately 40% in dKO versus CTR mice (Fig. 2H). To determine the reason for the lower NO level, eNOS activity in primary MLECs from CTR and dKO mice was measured. Sp1 and Sp3 deletion markedly attenuated the increased eNOS activity in MLECs (Fig. 2I). These data suggest that the impaired vasodilation in the dKO mesenteric resistance arteries was mediated by eNOS and Sp1/Sp3 play a crucial role in endothelial function.

Consistent with the phenotype of hypertension, we found a significant enlargement in hearts from dKO versus CTR littermates (Fig. 2J). We measured cardiac function using echocardiography. dKO mice showed a hypertrophic phenotype as indicated by the increased thickness of the diastolic left ventricular posterior wall and the systolic left ventricular posterior wall along with ejection fraction and fraction shortening (Fig. 1K–O). These results show that Sp1/Sp3 deficiency in endothelia resulted in elevated blood pressure, impaired vasodilation, and cardiac hypertrophy.

### Sp1 and Sp3 positively regulate AMPKα1 and AMPKα2 transcription

**Fig. 3:**
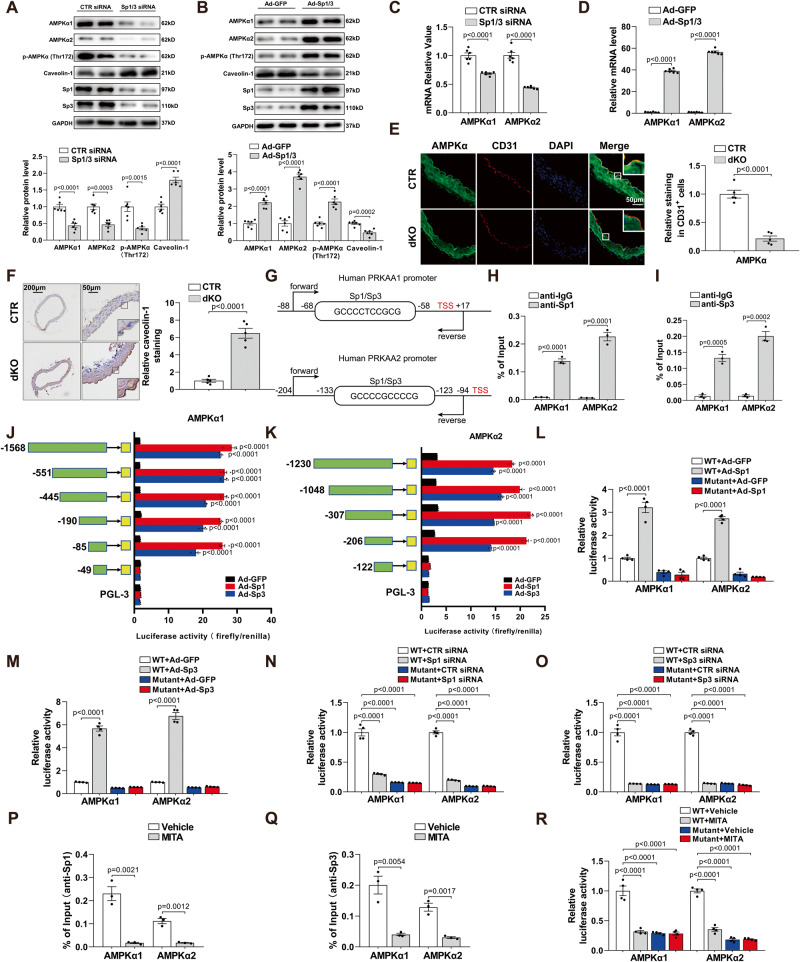
Sp1 and Sp3 positively regulate AMPKα1 and AMPKα2 transcription. **A** WB analysis and **C** qPCR analysis of AMPKα1, AMPKα2, p-AMPKα (Thr172), caveolin-1 in HUVECs transfected with CTR siRNA or Sp1/3 siRNA. *n* = 6. **B** WB analysis and **D** qPCR analysis of AMPKα1, AMPKα2, p-AMPKα (Thr172), caveolin-1 protein levels in HUVECs infected with Ad-GFP or Ad-Sp1/3. *n* = 6. **E** IF staining of AMPKα in aortas. CD31 is an endothelial cell marker. Scale bar: 50 μm. Quantitative analysis (right). *n* = 5. **F** Immunohistochemical (IHC) staining for caveolin-1 in aortas. Scale bar: 200 μm/50 μm. Quantitative analysis (right). *n* = 5. **G** Schematic illustration of putative Sp1/Sp3 binding region on the promoter of human *AMPKα1* and *AMPKα2*. Forward primer (forward) and reverse primer (reverse) for chromatin immunoprecipitation (ChIP) assay were indicated by arrows. ChIP assays showing the binding of Sp1 (**H**) or Sp3 (**I**) to the AMPKα1 and AMPKα2 promoters in HUVECs. *n* = 3. Luciferase activity shown by the indicated serial 5′ promoter deletions of AMPKα1 (**J**) and AMPKα2 (**K**) in HUVECs infected with Ad-GFP, Ad-Sp1, or Ad-Sp3. *n* = 4. **L**, **M** Relative luciferase activity for the wild-type (WT) and mutant constructs of AMPKα1 and AMPKα2 promoter in HEK293T cells infected with Ad-GFP, Ad-Sp1 (**L**) or Ad-Sp3 (**M**). *n* = 4. **N**, **O** Relative luciferase activity for the WT and mutant constructs of AMPKα1 and AMPKα2 promoter in HUVECs transfected with CTR, Sp1 (**N**) or Sp3 siRNA (**O**). *n* = 4. ChIP assays in HUVECs with or without mithramycin (MITA) using anti-Sp1 (**P**) or anti-Sp3 (**Q**) *n* = 3. **R** Relative luciferase activity for the WT and mutant constructs of AMPKα1 and AMPKα2 promoters in HUVECs with or without MITA. *n* = 4. Data are presented as mean ± SEM. Two-tailed Student unpaired *t* test for (**A**), (**B**), (**C**), (**D**), (**E**), (**F**), (**H**), (**I**), (**P**), and (**Q**). One-way ANOVA followed by Bonferroni post hoc analysis for (**J**), (**K**), (**L**), and (**M**). Two-way ANOVA followed by Bonferroni post hoc test for (**N**), (**O**), and (**R**).

To explore the specific mechanism of Sp1/Sp3 ablation causing endothelial dysfunction and hypertension, we used transcriptomic analysis of MLECs from CT R and dKO mice and identified 2683 genes with altered expression upon Sp1/Sp3 deletion (1591 upregulated and 1092 downregulated, *p* < 0.05 and |Log2FoldChange| > 1.5) (Fig. S2A). We further determined the expression of specific genes via knockdown and overexpression studies. Levels of phosphorylated eNOS at Ser1177 and total eNOS did not differ between in CTR and dKO MAECs (Fig. S2B). Being the key molecule that can bind to eNOS and inhibit its bioactivity in endothelial cells, caveolin-1 showed a significant increase in expression in dKO MLECs (Fig. S2B). Co-immunoprecipitation (CoIP) of caveolin-1 and eNOS increased in dKO MLECs (Fig. S2C, D), which suggests that Sp1/Sp3 ablation promoted caveolin-1 binding with eNOS and reduced eNOS activity. Knockdown of Sp1 and Sp3 by siRNA in HUVECs downregulated AMPKα1, AMPKα2, and p-AMPKα (Thr172) protein levels (Fig. 3A) and AMPKα1/AMPKα2 mRNA levels (Fig. 3C), but upregulated caveolin-1 protein levels (Fig. 3A). In contrast, transient overexpression of Sp1 and Sp3 in HUVECs significantly reduced caveolin-1 level and increased AMPKα1, AMPKα2, and p-AMPKα (Thr172) protein levels (Fig. 3B). Similar results were found in MLECs from CTR and dKO mice (Fig. S2B). Furthermore, the mRNA levels of AMPKα1 and AMPKα2 were upregulated by Sp1/Sp3 overexpression in HUVECs (Fig. 3D). IF staining further confirmed significantly decreased protein level of AMPKα in the aortic endothelium of dKO mice (Fig. 3E). The level of caveolin-1 protein expression was markedly elevated in the aortic endothelial layers of dKO mice as detected using immunohistochemical (IHC) staining (Fig. 3F).

To verify whether Sp1 and Sp3 could interact with the AMPKα1 and AMPKα2 promoters, we used chromatin immunoprecipitation (ChIP) assay. ChIP with antibodies against Sp1 or Sp3 were cross-linked to the binding site cluster, therefore both Sp1 and Sp3 could bind with predicted AMPKα1 (−68/−58 bp) and AMPKα2 (−133/−123 bp) promoters (Fig. 3G–I).

To further understand the transcriptional regulation of AMPKα1 and AMPKα2 by Sp1 and Sp3, we cloned the different truncated promoters of AMPKα1 and AMPKα2 upstream of the luciferase gene to generate truncation promoter reporters. The observed AMPKα1 promoter activity isolated a minimal Ad-Sp1- and Ad-Sp3-responsive element when compared to pGL-3 at the −85 to −49 segment (Fig. 3J), which suggests a role for this core region in regulating AMPKα1 transcription. To verify the putative transcription factor binding site for Sp1 and Sp3 in the AMPKα1 promoter, we prepared a substitution mutation of this individual site, which resulted in a significant decrease in transcriptional activity when compared to the wild-type promoter (Fig. 3L, M). To validate the regulatory roles of Sp1 and Sp3 in the AMPKα2 promoter, deletion constructs showed a prominent increase in promoter activity with Ad-Sp1 or Ad-Sp3 administration, except for in the −206 to −122 segment, which indicates a specific association of Sp1 and Sp3 with this region (Fig. 3K). Furthermore, HEK293T cells transfected with a mutated fragment construct showed lower luciferase activity relative to cells transfected with the wild-type promoter (Fig. 3L, M). In HUVECs, Sp1 or Sp3 knockdown by siRNA confirmed a marked decrease in luciferase activity of both wild-type AMPKα1 and AMPKα2, but not the mutant promoter constructs (Fig. 3N, O). Moreover, we used mithramycin A (MITA), a specific inhibitor of Sp1 transcription activation (which functions by blocking Sp1 and Sp3 binding to GC-rich regions) to further confirm the effect of Sp1 on AMPKα1 and AMPKα2 transcription. ChIP assays revealed that MITA could repress Sp1 binding with AMPKα1 and AMPKα2 promoters (Fig. 3P, Q). Luciferase activity was also reduced in MITA-treated HUVECs transfected with wild-type AMPKα1 or AMPKα2, but not the mutant promoter constructs (Fig. 3R). These results demonstrate that Sp1/Sp3 could directly bind to the AMPKα1 and AMPKα2 promoters and promote their transcriptional expression.

### Sp1 and Sp3 improve endothelial function via AMPKα

**Fig. 4:**
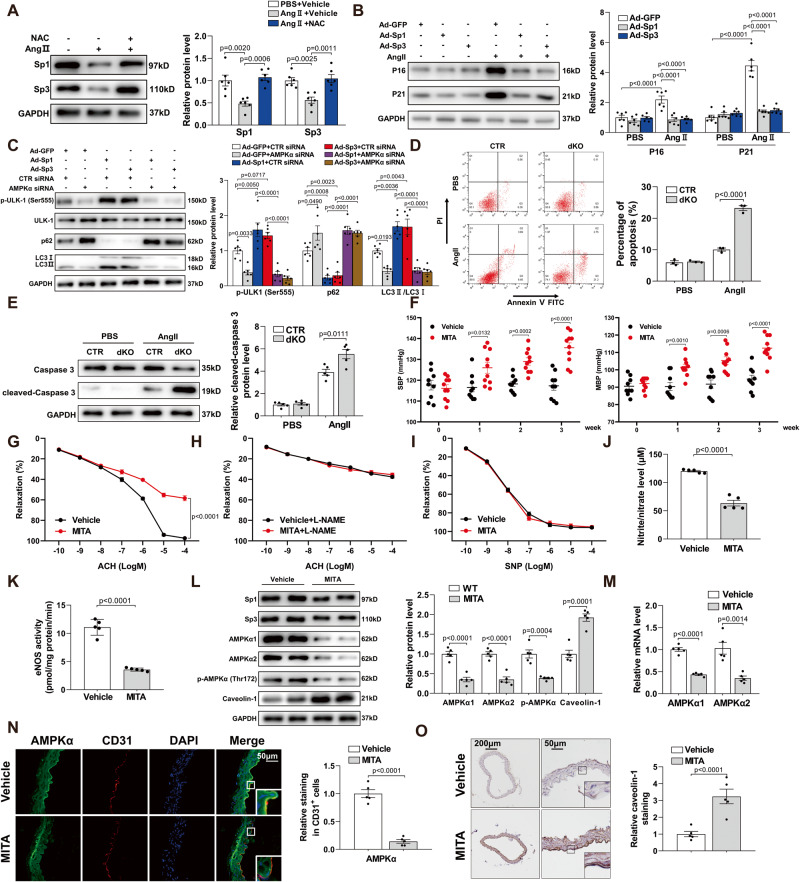
Sp1 and Sp3 improve endothelial function via AMPKα. **A** WB of Sp1 and Sp3 protein levels in HUVECs with different treatments. *n* = 6. **B** WB of P16 and P21 protein levels in HUVECs with different treatments. *n* = 6. **C** WB of p-ULK1 (Ser555), ULK1, p62, and LC3 II/LC3 I in HUVECs with different treatments. *n* = 6. **D** MLECs from CTR and dKO mice with different treatments were stained with fluorescein isothiocyanate (FITC)-conjugated annexin V and propidium iodide (PI) and analyzed by flow cytometry. *n* = 3. **E** WB of caspase 3 and cleaved-caspase 3 protein levels in MLECs from CTR and dKO mice with different treatments. *n* = 5. **F** SBP and MBP in mice treated with vehicle or MITA. *n* = 9−10. **G**–**I** Vascular reactivity of mesenteric arteries from vehicle- or MITA-treated mice to Ach with or without L-NAME pretreatment (10^–4^ mol/L, 30 min) and SNP. *n* = 5. **J** Serum nitrite/nitrate level from vehicle- and MITA-treated mice. *n* = 5. **K** eNOS activity measured in MLECs from vehicle- and MITA-treated mice. *n* = 5. **L** WB of protein levels of Sp1, Sp3, AMPKα1, AMPKα2, p-AMPKα (Thr172), and caveolin-1 in MLECs from vehicle- and MITA-treated mice. *n* = 5. **M** qPCR analysis of AMPKα1 and AMPKα2 mRNA levels in MLECs from vehicle and MITA-treated mice. *n* = 5. **N** Representative IF staining of AMPKα in aortas from vehicle and MITA-treated mice. CD31 is an endothelial cell marker. Scale bar: 50 μm. Quantitative data analysis (right). *n* = 5. **O** Representative IHC staining for caveolin-1 in aortas from vehicle and MITA-treated mice, Scale bar: 200 μm/50 μm. Quantitative data analysis (right). *n* = 5. Data are presented as mean ± SEM. Two-tailed Student unpaired *t* test for (**D**), (**E**), (**F**), (**J**), (**K**), (**L**), (**M**), (**N**), and (**O**). One-way ANOVA followed by Bonferroni post hoc analysis for (**A**), (**B**), and (**C**). Two-way ANOVA followed by Bonferroni post hoc test for (**G**) to (**I**).

To further explore the molecular mechanism by which Sp1 and Sp3 regulate endothelial function, we used Ang II, implicated in the pathogenesis of endothelial dysfunction, as a representative stimulus. The activity of the antioxidant N-acetyl-L-cysteine (NAC) decreased the protein levels of Sp1 and Sp3 induced by Ang II (Fig. 4A), which suggests that Ang II aggravated endothelial dysfunction by promoting reactive oxygen species (ROS) and reducing Sp1 and Sp3 expression. Overexpression of Sp1 or Sp3 markedly ameliorated Ang II-induced endothelial senescence, as indicated by the reduced expression of P16 and P21, which are markers of endothelial cell senescence (Fig. 4B). Knockdown of AMPKα by siRNA in HUVECs decreased the levels of p-ULK1 (Ser555) and microtubule-associated protein 1 light chain 3 (LC3) II/I ratio induced by Sp1 and Sp3 and increased the expression of p62 (Fig. 4C); AMPKα therefore plays a vital role in Sp1/Sp3-induced autophagy. Moreover, Ang II induced more severe apoptosis in Sp1/Sp3-deficient MLECs than the CTR mice as detected using flow cytometry (Fig. 4D) and a western blot assay (Fig. 4E). Thus, Sp1 and Sp3 could improve endothelial function via AMPK. Furthermore, Ang II, ET-1, and H_2_O_2_ reduced the Sp1/Sp3 association with AMPKα1 and AMPKα2 promoters (Fig. S3A, B).

To further confirm whether the suppression of Sp1 and Sp3 could cause endothelial dysfunction, C57BL/6 J mice were chronically treated with the anti-carcinogen MITA for 3 weeks. SBP and MBP were higher in MITA-treated mice than in wild-type mice (Fig. 4F). Administration of MITA impaired endothelium-dependent vasorelaxation (Fig. 4G–I) and lowered the serum nitrite/nitrate level (Fig. 4J) owing to decreased eNOS activity (Fig. 4K); this suggests that the endothelial dysfunction was exacerbated by MITA in vivo.

IF staining showed a significant decrease in AMPKα content in the aortic endothelium treated with MITA (Fig. 4N). Moreover, WB revealed a MITA-mediated decrease in AMPKα1, AMPKα2, and p-AMPKα (Thr172) protein levels leading to increased caveolin-1 expression in MLECs from MITA-treated mice (Fig. 4L); this was also confirmed by IHC staining (Fig. 4O). qPCR assays revealed that MITA infusion reduced the mRNA levels of AMPKα1 and AMPKα2 (Fig. 4M), which suggests that MITA represses AMPKα1/AMPKα2 transcription by inhibiting Sp1/Sp3 activity. IP assays were performed to further determine the inhibitory effect of MITA on caveolin-1 interacting with eNOS in MLECs from MITA-treated mice (Fig. S4A, B). Flow cytometry analysis and WB assays revealed that MITA exacerbated apoptosis induced by Ang II (Fig. S4C, D). These data indicate that the inhibition of Sp1 and Sp3 impaired endothelial function, which is consistent with the findings in dKO mice.

### Administration of CA-AMPK decreases blood pressure and alleviates endothelial dysfunction in Sp1/Sp3^ECKO^ mice

**Fig. 5:**
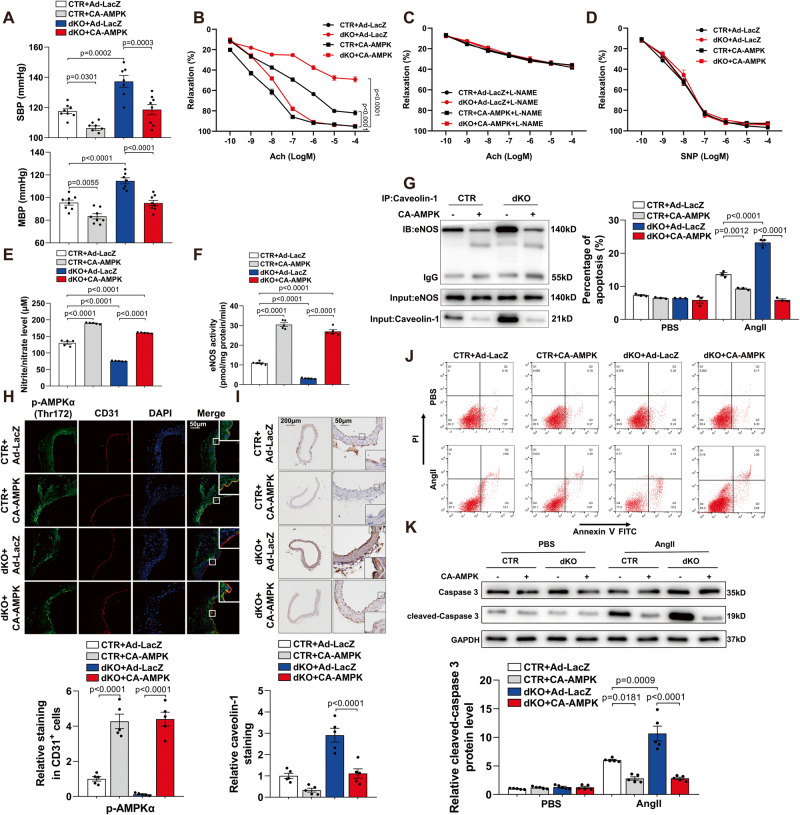
Administration of CA-AMPK decreases blood pressure and alleviates endothelial dysfunction in Sp1/Sp3^ECKO^ mice. **A** SBP and MBP in CTR and dKO mice with Ad-LacZ or CA-AMPK. *n* = 7–8. **B**–**D** Vascular reactivity of mesenteric arteries from CTR and dKO mice with Ad-LacZ or CA-AMPK to Ach with or without L-NAME pretreatment (10^–4^ mol/L, 30 min) and SNP. *n* = 5. **E** Serum nitrite/nitrate level from CTR and dKO mice with Ad-LacZ or CA-AMPK. *n* = 5. **F** eNOS activity measured in MLECs from CTR and dKO mice with Ad-LacZ or CA-AMPK. *n* = 5. **G** Co-immunoprecipitation (CoIP) assay of eNOS immunoprecipitated with anti-caveolin-1 antibody in MLECs from CTR and dKO mice with Ad-LacZ or CA-AMPK. *n* = 5. **H** Representative IF staining of p-AMPKα (Thr172) in aortas from CTR and dKO mice with Ad-LacZ or CA-AMPK. CD31 is an endothelial cell marker. Scale bar: 50 μm. Quantitative data analysis (below). *n* = 5. **I** Representative IHC staining for caveolin-1 in aortas from CTR and dKO mice with Ad-LacZ or CA-AMPK, Scale bar: 200 μm/50 μm. Quantitative data analysis (below). *n* = 5. **J** MLECs from CTR and dKO mice with Ad-LacZ or CA-AMPK with different treatments were stained with FITC-conjugated annexin V and PI and analyzed by flow cytometry. *n* = 3. **K** WB of caspase 3 and cleaved-caspase 3 protein levels in MLECs from CTR and dKO mice with Ad-LacZ or CA-AMPK with different treatments. *n* = 5. Data are presented as mean ± SEM. One-way ANOVA followed by Bonferroni post hoc analysis for (**A**), (**E**), (**F**), (**H**), (**I**), (**J**), and (**K**). Two-way ANOVA followed by Bonferroni post hoc test for (**B**) to (**D**).

To determine the role of AMPK in Sp1/Sp3-mediated endothelial function, CTR and dKO mice were injected with adenovirus expressing constitutively active (CA)-AMPK. The administration of Ad-CA-AMPK increased AMPKα in vascular endothelial cells in CTR and dKO mice. The SBP and MBP of CTR and dKO mice decreased after 7 days, but the administration of Ad-LacZ had no effect on blood pressure (Fig. 5A). Ach-induced endothelial relaxation responses in both, CTR and dKO mice were ameliorated following CA-AMPK administration (Fig. 5B). CA-AMPK could not ameliorate endothelium-dependent relaxation blocked by L-NAME and had no effect on endothelium-independent relaxation caused by SNP (Fig. 5C, D). Moreover, CA-AMPK administration greatly increased serum nitrite/nitrate level and eNOS activity in control Sp1/Sp3-deficient MLECs (Fig. 5E, F). Furthermore, CA-AMPK reduced eNOS binding with caveolin-1 in MLECs from CTR and dKO mice (Fig. 5G).

The CA-AMPK-increased p-AMPKα (Thr172) level in endothelial cells of CTR and dKO mice was confirmed by IF staining with CD31 (Fig. 5H). Consistently, caveolin-1 expression in the aortic endothelium decreased with CA-AMPK treatment (Fig. 5I). MLEC apoptosis induced by Ang II was eliminated by CA-AMPK administration (Fig. 5J, K), which indicates an essential role of AMPKα regulated by Sp1 and Sp3 in Ang II-induced endothelial apoptosis. Our data indicates that AMPKα plays an important role in the Sp1/Sp3-mediated regulation of endothelial function and blood pressure.

### Endothelial Sp1 and Sp3 are implicated in the effects of captopril

**Fig. 6:**
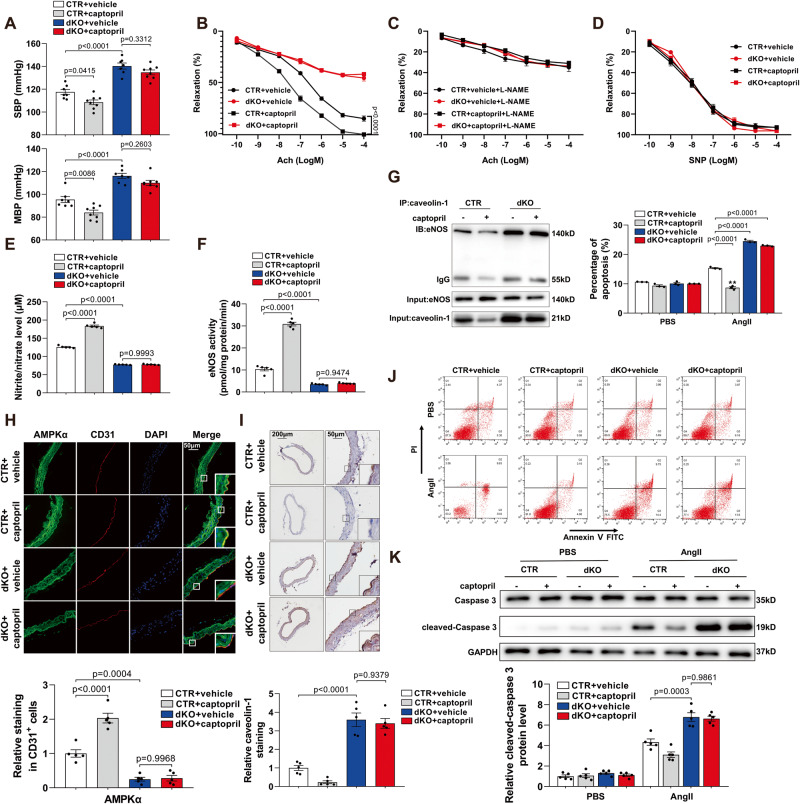
Endothelial Sp1 and Sp3 are responsible for anti-hypertension and anti-endothelial dysfunction effects of captopril. **A** SBP and MBP in CTR and dKO mice treated with vehicle or captopril. *n* = 7–8. **B**–**D** Vascular reactivity of mesenteric arteries from CTR and dKO mice treated with vehicle or captopril to Ach with or without L-NAME pretreatment (10^–4^ mol/L, 30 min) and SNP. *n* = 5. **E** Serum nitrite/nitrate level from CTR and dKO mice with vehicle or captopril. *n* = 5. **F** eNOS activity measured in MLECs from CTR and dKO mice with vehicle or captopril. *n* = 5. **G** CoIP assay of eNOS immunoprecipitated with anti-caveolin-1 antibody in MLECs from CTR and dKO mice with vehicle or captopril. *n* = 5. **H** Representative IF staining of AMPKα in aortas from CTR and dKO mice with vehicle or captopril. CD31 is an endothelial cell marker. Scale bar: 50 μm. Quantitative data analysis (below). *n* = 5. **I** Representative IHC staining for caveolin-1 in aortas from CTR and dKO mice with vehicle or captopril, Scale bar: 200 μm/50 μm. Quantitative data analysis (below). *n* = 5. **J** MLECs from CTR and dKO mice with vehicle or captopril with different treatments were stained with FITC-conjugated annexin V and PI and analyzed by flow cytometry. *n* = 3. **K** WB analysis of caspase 3 and cleaved-caspase 3 protein levels in MLECs from CTR and dKO mice with vehicle or captopril with different treatments. *n* = 5. Data are presented as mean ± SEM. One-way ANOVA followed by Bonferroni post hoc analysis for (**A**), (**E**), (**F**), (**H**), (**I**), (**J**), and (**K**). Two-way ANOVA followed by Bonferroni post hoc test for (**B**) to (**D**).

To determine whether ACEI improves endothelial dysfunction and lowers blood pressure via Sp1/Sp3, CTR and dKO mice were intraperitoneally administered captopril. Captopril lowered blood pressure and improved endothelium-dependent relaxation of mesenteric resistance arteries from CTR, but not dKO mice (Fig. 6A–D). Captopril increased the serum nitrite/nitrate level by enhancing eNOS activity in CTR mice, but not in dKO mice (Fig. 6E, F). Consistently, binding of eNOS decreased with caveolin-1 in MLECs from captopril-treated CTR, but not dKO mice (Fig. 6G). IF staining confirmed an increased level of AMPKα in aortas from captopril-treated CTR mice, with no change in the dKO mice after captopril treatment (Fig. 6H). Moreover, IHC analysis revealed reduced caveolin-1 protein levels regulated by captopril treatment in aortas from CTR versus dKO mice (Fig. 6I). Furthermore, captopril reduced Ang II-induced apoptosis in CTR MLECs. but had no effect on Sp1/Sp3-deficient MLECs treated with Ang II (Fig. 6J, K).

To further investigate the broad implications of Sp1/Sp3 signaling and captopril in hypertension, Ang II-induced hypertensive mice were studied. After Ang II treatment for 4 weeks, SBP, MBP, and diastolic blood pressure (DBP) levels in dKO mice were higher by approximately 20–30 mmHg when compared with CTR mice (Fig. S5A–C). In addition, Ang II treatment significantly exacerbated the endothelial-dependent vasorelaxation of mesenteric arteries from dKO mice compared with Ang II + CTR mice (Fig. S5D–F). Pretreatment with captopril lowered blood pressure and improved endothelium-dependent relaxation of mesenteric resistance arteries from CTR, but not dKO mice (Fig. S5A–F). Taken together, these data indicate that Sp1 and Sp3 deletion abolished the captopril-induced alleviation of endothelial dysfunction and the anti-apoptotic effects of captopril in endothelial cells; Sp1 and Sp3 have an indispensable role in the success of captopril treatment in the endothelium.

### Captopril activates Sp1 and Sp3 via bradykinin B1 receptor

**Fig. 7:**
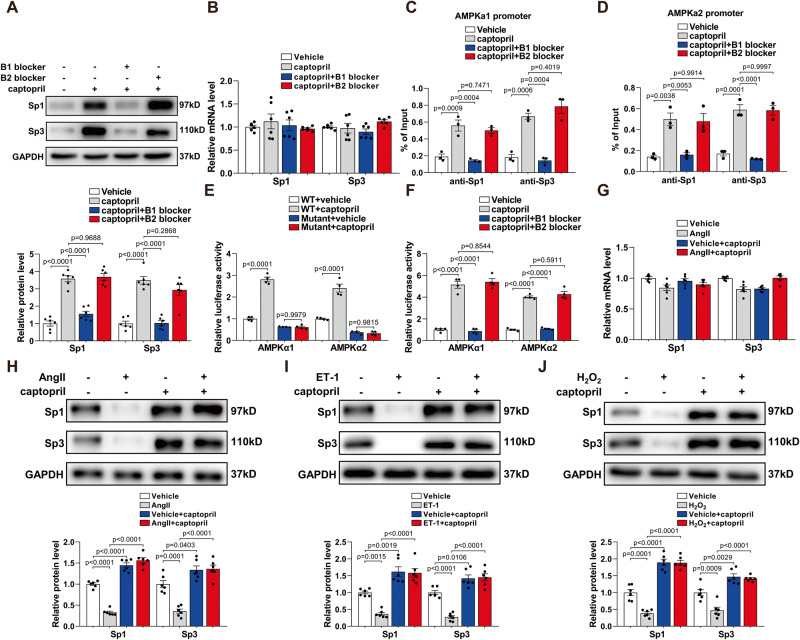
Captopril activates Sp1 and Sp3 via bradykinin B1 receptor. **A** WB analysis of Sp1 and Sp3 protein levels in HUVECs with different treatments. *n* = 6. **B** qPCR analysis of Sp1 and Sp3 mRNA levels in HUVECs with different treatments. *n* = 6. **C**, **D** ChIP assays showing the binding of Sp1 or Sp3 to the AMPKα1 (**C**) and AMPKα2 (**D**) promoter in HUVECs with different treatments. *n* = 3. **E** Relative luciferase activity for the WT and mutant constructs of AMPKα1 and AMPKα2 promoter in HUVECs with vehicle or captopril. *n* = 4. **F** Relative luciferase activity for the WT constructs of AMPKα1 and AMPKα2 promoter in HUVECs with different treatments. *n* = 4. **G** qPCR analysis of Sp1 and Sp3 mRNA levels in HUVECs with different treatments. *n* = 3. **H**–**J** WB analysis of Sp1 and Sp3 protein levels in HUVECs with different treatments. *n* = 6. Data are presented as mean ± SEM. Two-way ANOVA followed by Bonferroni post hoc analysis for (**A**) to (**J**).

Previous studies have suggested that nanomolar concentrations of ACEI directly activate the bradykinin B1 receptor in cells in the absence of ACE or bradykinin, thus resulting in increased generation of NO^23^. To investigate whether captopril could directly bind to the bradykinin B1 receptor to activate Sp1 and Sp3, we tested it in HUVECs using des-Arg9-[Leu8]-bradykinin (B1 blocker) or fasitibant chloride hydrochloride (B2 blocker). The presence of B1 receptor was demonstrated in MLECs (Fig. S6A) and HUVECs, showing the same level with or without captopril (Fig. S6B). Sp1 and Sp3 protein levels were increased by captopril and decreased after treatment with B1 blocker, but not B2 blocker (Fig. 7A). Captopril did not alter Sp1 and Sp3 mRNA levels (Fig. 7B). A ChIP assay indicated that captopril increased Sp1 and Sp3 binding with the AMPKα1 and AMPKα2 promoters, which was inhibited by B1 blocker (Fig. 7C, D). To further verify the role of captopril, AMPKα1 and AMPKα2 luciferase constructs were significantly activated in the presence of captopril (Fig. 7E, F), but this effect was abolished by mutations of the Sp1/Sp3 binding site (Fig. 7E) or by B1 blocker treatment (Fig. 7F). These results suggest that the B1 receptor is the predominant receptor contributing to captopril-induced AMPKα1/AMPKα2 transcription via Sp1 and Sp3. Furthermore, we determined whether captopril could increase Sp1 and Sp3 levels under pathological conditions in vitro. Captopril treatment reversed the reduced levels of Sp1 and Sp3 caused by Ang II, ET-1, and H_2_O_2_ (Fig. 7H–J) but had no effect on Sp1 and Sp3 mRNA levels (Fig. 7G).

As shown in Fig. S6, mesenteric arteries dissected from Ang II-induced hypertensive CTR mice were preincubated for 6 h separately with vehicle, captopril, lisinopril (ACEI that does not stimulate B1R), ARB, captopril + B1 blocker, or captopril + B2 blocker. Compared with vehicle, captopril significantly improved Ach-induced endothelium-dependent vasorelaxation (Fig. S6C). In contrast, mesenteric arteries preincubated with lisinopril or ARB exhibited similar vascular function to the vehicle group. In addition, B1 but not B2 blocker attenuated the effect of captopril on endothelium-dependent vasorelaxation, and therefore inhibited alleviation of this vasorelaxation (Fig. S6C). Almost all the endothelium-dependent vasorelaxation responses were blocked by L-NAME (Fig. S6D). There was no difference in the endothelium-independent vasorelaxation responses induced by SNP in mesenteric resistance arteries isolated from these groups of mice (Fig. S6E). These data support the conclusion that B1 agonism function contributes toward the anti-endothelial dysfunction of captopril.

Inhibition of Sp1 and Sp3 by MITA in bovine aortic endothelial cells (BAECs) downregulated NO production and eNOS activity. Inhibition of Sp1 and Sp3 eliminated the captopril-induced increase in NO production and eNOS activity (Fig. S6F, G). In addition, treatment with captopril in the absence of bradykinin in the medium caused an increase in NO production and eNOS activity (Fig. S6H, I). In addition, B1 blocker attenuated this effect of captopril. The data showed a tendency for the B2 blocker to block captopril’s effect in the absence of bradykinin but did not reach statistical significance (Fig. S6H, I).

### HDAC1-mediated Sp1/Sp3 deacetylation is responsible for captopril-induced AMPKα1/AMPKα2 transcription activation

**Fig. 8:**
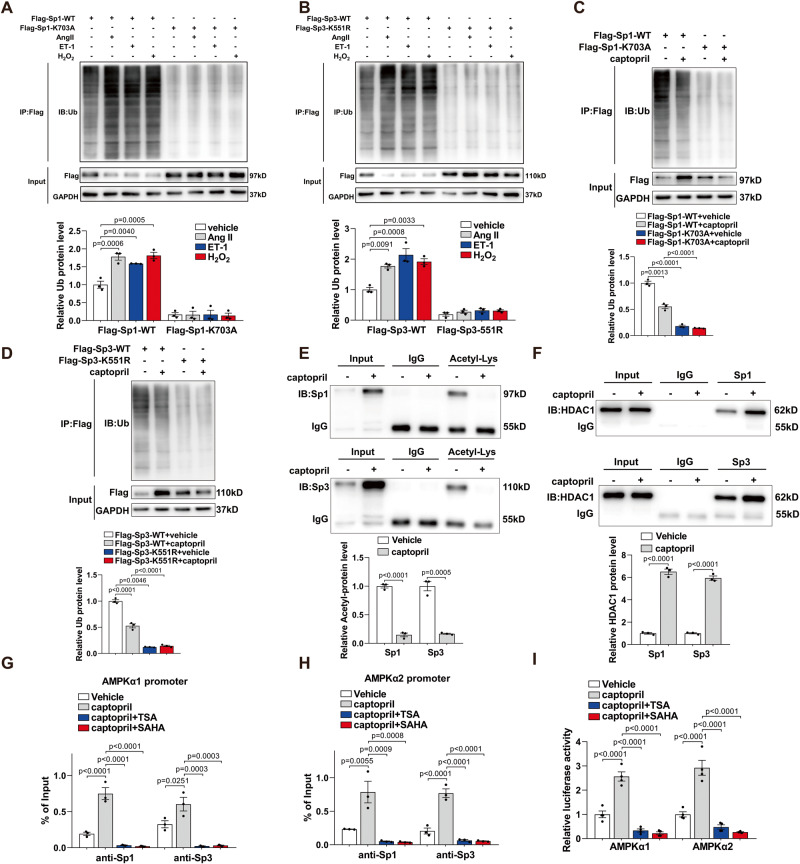
HDAC1-mediated Sp1/Sp3 deacetylation is responsible for captopril-induced AMPKα1/AMPKα2 transcription activation. **A** CoIP assay of Sp1 ubiquitination in HUVECs transfected with Flag-Sp1-WT or Flag-Sp1-K703A with different treatments. Quantified analysis of ubiquitin protein level (below). *n* = 3. **B** Co-IP analysis of Sp3 ubiquitination in HUVECs transfected with Flag-Sp3-WT or Flag-Sp3-K551R with different treatments. Quantified analysis of ubiquitin protein level (below). *n* = 3. **C**, **D** CoIP assay of **C** Sp1 and **D** Sp3 ubiquitination in HUVECs treated with captopril. Quantified analysis of ubiquitin protein level (below). *n* = 3. **E** CoIP assay of acetylation levels of Sp1 or Sp3 in HUVECs treated with captopril. Quantified analysis of acetyl-protein level (below). *n* = 3. **F** CoIP assay of HDAC1 in HUVECs treated with captopril. Quantified analysis of HDAC1 protein level (below). *n* = 3. **G**, **H** ChIP assay showing the binding of Sp1 or Sp3 to the AMPKα1 (**G**) and AMPKα2 (**H**) promoter in HUVECs with different treatments. *n* = 3. **I** Relative luciferase activity for the WT constructs of AMPKα1 and AMPKα2 promoter in HUVECs with different treatments. *n* = 4. Data are presented as mean ± SEM. Two-tailed Student unpaired *t* test for (**E**) and (**F**). One-way ANOVA followed by Bonferroni post hoc analysis for (**G**–**I**). Two-way ANOVA followed by Bonferroni post hoc test for (**A**–**D**).

The above results indicated that post-translational modification of Sp1/Sp3 is involved in the captopril-increased and Ang II/ET-1/H_2_O_2_-suppressed Sp1/Sp3 protein levels. The three ways to promote protein degradation are autophagy, proteasome regulation, and via lysosomal pathways. HUVECs were pretreated with the autophagy inhibitor 3-MA, ubiquitin-proteasome inhibitor MG132, or lysosomal inhibitor CQ to explore the Sp1/Sp3 degradation pathway. Ang II/ET-1/H_2_O_2_-induced Sp1/Sp3 degradation could be attenuated by MG132 but not 3-MA or CQ (Fig. S7A), which suggests that Ang II/ET-1/H_2_O_2_ promoted Sp1/Sp3 degradation via the ubiquitin-proteasome pathway. Sp1/Sp3 were significantly poly-ubiquitinated by Ang II/ET-1/H_2_O_2_ (Fig. 8A, B). In contrast, captopril could reduce the poly-ubiquitination of Sp1/Sp3 (Fig. 8C, D). Acetylation is known to promote protein degradation by elevating poly-ubiquitination^24,25^. To investigate the possible role of the acetylated Lys-703 residue of Sp1 in regulating Sp1 ubiquitination, HUVECs were transfected with Flag-Sp1-WT or Flag-Sp1-K703A of which the Lys-703 residue was mutated to alanine. As compared with Flag-Sp1-WT, the mutated Flag-Sp1-K703A protein had a low basal level of ubiquitination, which did not respond to captopril or Ang II/ET-1/H_2_O_2_ (Fig. 8A, C). Furthermore, we observed similar results in HUVECs transfected with plasmids carrying Flag-Sp3-WT or Flag-Sp3-K551R of which the Lys-551 residue was mutated to arginine (Fig. 8B, D). These results indicate that Sp1/Sp3 ubiquitination depends on acetylation.

We then measured the protein expression of acetylated Sp1/Sp3 by immunoprecipitating Sp1/Sp3 antibody and probing with anti-pan-acetyl-lysine antibody. Sp1/Sp3 acetylation significantly reduced in HUVECs after captopril treatment (Fig. 8E) and increased after Ang II/ET-1/H_2_O_2_ treatment (Fig. S7B). Further, to address whether captopril recruits the histone deacetylase 1 (HDAC1), we analyzed the association of HDAC1 with Sp1/Sp3 using the CoIP assay in HUVECs treated with captopril. Sp1 and Sp3 could combine more HDAC1 protein in the captopril as compared with the non-captopril condition (Fig. 8F), which suggests that captopril induces HDAC1 recruitment of Sp1/Sp3, resulting in decreased Sp1/Sp3 acetylation. To further explore the link between acetylation and degradation of Sp1/Sp3, we used a knockdown of HDAC1 and inhibitor treatment with trichostatin A (TSA) and vorinostat (SAHA). Knockdown of HDAC1 reversed the increased protein levels of Sp1/Sp3 induced by captopril and greatly increased the ubiquitination of Sp1/Sp3 (Fig. S7C), which was consistent with the results with TSA or SAHA treatment (Fig. S7D). Moreover, captopril induced more binding of Sp1/Sp3 with AMPKα1 and AMPKα2 promoters, which was suppressed by the HDAC1 inhibitor TSA or SAHA (Fig. 8G, H). Evaluation of luciferase activity revealed results were similar to those with the ChIP assay (Fig. 8I). To further investigate the changing affinity of Sp1/Sp3 to the promoters in chromatin context, we performed a series of ChIP assays. Captopril significantly increased HDAC1 and HDAC2 binding to the promoters but had no effect on HDAC6 (Fig. S8A, B). In contrast, no changes were observed in the classical acetyltransferase p300/CBP binding to the promoters (Fig. S8C, D). Captopril enhanced the Flag-Sp1-WT or Flag-Sp3-WT binding to the AMPKα1 and AMPKα2 promoters. However, the mutated Sp1 and Sp3 exhibited lesser affinity to the promoters and no response to captopril (Fig. S8E, F). The reduction of Sp1/Sp3 with siRNA Sp1/Sp3 or overexpression with Ad-Sp1/Sp3 also changed their affinity to the promoters, confirming that the specific recruitment of factors to a particular promoter actually responds to the factor level (Fig. S9A–H). Collectively, HDAC1-induced Sp1/Sp3 deacetylation was responsible for captopril-induced AMPKα1/AMPKα2 transcription activation.

To further verify the role of the bradykinin B1 receptor in signaling, we used Des-Arg9-Bradykinin (a selective B1 receptor agonist) to directly activate the B1 receptor, instead of captopril. Similar results were also found in Des-Arg9-Bradykinin-treated HUVECs (Fig. S10).

## Discussion

Hypertension is the leading preventable risk factor for cardiovascular disease, which is the greatest contributor to all-cause mortality worldwide^26^. Due to the widespread use of antihypertensive medications, the global MBP has remained constant or decreased slightly over the past 4 decades^27^. Hypertension has been linked to deficient NO levels and increased vascular production of ROS caused by endothelial dysfunction^28^. It is imperative that we explore the mechanisms underlying hypertension and identify potential biomarkers and strategies for its prevention and treatment. In this study, we demonstrated that endothelial-specific Sp1/Sp3 deletion resulted in endothelial dysfunction and hypertension, and that captopril, an ACE inhibitor and traditional antihypertensive drug, exerted part of its pharmacological effect by modulating Sp1/Sp3 levels in endothelial cells.

Characterized by three conserved zinc fingers, Sp1 and Sp3 are widely expressed in mammalian cell types as transcription activators or suppressors. They bind with the same affinity and specificity to the GC box or closely related GT box in the regulator regions of numerous genes^29,30^. Sp1-deficient mouse embryos are severely retarded in development, showing a wide range of defects, and die on approximately day 11 of gestation^11^. Sp3-deletion mouse embryos showed a range of severe cardiac malformations causing damage to circulation at embryonic day 14.5^12^. In our prior investigation, we have generated inducible endothelium-specific Sp1/Sp3-knockout mice, by breeding *Sp1*^*fl/fl*^*/Sp3*^*fl/fl*^ with *VE-CAD-CreER*^*T2*^ mice^15^. The loss of Sp1/Sp3 delayed the angiogenesis in neonatal retinas, reparative angiogenesis to hindlimb ischemia and skin wound, even the angiogenesis in the subcutaneous tumor. From previous studies, we suspected that Sp1 and Sp3 played an important role in the pathogenesis of cardiovascular diseases. Indeed, the dKO mice reproduced some features of human hypertension including decreased eNOS activity, endothelial dysfunction, and cardiac hypertrophy. Sp1 and Sp3 were downregulated in aortic endothelial cells from Ang II-induced hypertensive mice and in mesenteric aortic endothelial cells from hypertensive patients. Ang II, ET-1, or H_2_O_2_-mediated Sp1/Sp3 downregulation in endothelial cells may be a common mechanism in the early development of hypertension. This study demonstrated that Sp1/Sp3 protect against endothelial dysfunction and hypertension, enhancing our understanding of the functions of Sp1 and Sp3.

AMPK, a highly conserved serine/threonine protein kinase involved in regulating cellular and whole organ metabolism, contains α, β, and γ subunits, each with two or more isoforms^31^. AMPK in endothelial cells has been implicated in the regulation of glucose metabolism^32^, lipid metabolism^33^, nitric oxide production^33^, atherosclerosis^34^, and angiogenesis^35^. In the endothelial cell layers in the aortas of AMPKα1^−/−^ or AMPKα2^−/−^ mice, caveolin-1 levels were markedly elevated, which decreased eNOS activity when compared to wild-type mice^9^. AMPK regulates eNOS activity via HuR-mediated caveolin-1, which inhibits eNOS as a “clamp” instead of changing its phosphate level^9^. In this study, eNOS activity and eNOS-dependent vasodilation were severely impaired in dKO mice, but the p-eNOS level was similar to that of CTR mice. To gain an insight into the possible reason behind these phenomena, we explored the link between Sp1/Sp3 and AMPKα. Sp1/Sp3 directly bound to the predicted sites in the AMPKα1 and AMPKα2 promoter regions crucial for transcription activation; this binding was inhibited by MITA. MITA is an anti-carcinogenic compound in various tumors due to its capacity to inhibit mRNA synthesis^36^. It binds preferentially to GC-rich regions of gene promoters, thus preventing binding of the transcription factors Sp1/Sp3^37^. However, we demonstrated that intraperitoneal administration of MITA repressed Ach-induced vasodilation and severe endothelial dysfunction in C57BL/6J mice, which highlights its cardiovascular toxicity and limits its clinical application. Here, we identified Sp1/Sp3 as the transcription factors of AMPKα1 and AMPKα2. Our results enrich the understanding of the regulatory mechanism of AMPKα expression.

Endothelial dysfunction including senescence, apoptosis, and impaired vasodilation plays a vital role in the development of cardiovascular diseases. Gene silencing or inhibition of Sp1/Sp3 led to severe endothelial dysfunction and hypertension, and overexpression of Sp1/Sp3 ameliorated endothelial senescence and apoptosis induced by Ang II; this suggests that Sp1/Sp3 may represent potential targets for drug design to treat cardiovascular diseases. Indeed, endothelial Sp1 and Sp3 were responsible for the anti-hypertension and anti-endothelial dysfunction effects of captopril, which is the major finding of this study. Most ACEIs, including captopril, have a region containing the ‘special sequence,’ which is crucial for binding with the B1 receptor^38^. Our in vivo results showed that captopril improved eNOS activity, enhanced endothelium-dependent vasodilation, and alleviated the apoptotic effects of Ang II in CTR versus dKO mice. This suggests that Sp1/Sp3 are likely downstream targets of captopril. Recent studies suggested that captopril could increase phosphorylation of Thr172 in AMPKα in primary neuron cells^39^ and in WT mice hearts^40^. In our study, we found that captopril could decrease Sp1/Sp3 acetylation and increase Sp1/Sp3 protein levels, thus increasing AMPKα1/AMPKα2 transcription and improving endothelial function; this was abolished by B1 receptor blockage. Mechanistically, acetylated Sp1/Sp3 were ubiquitinylated and degraded by proteasomes, and this could be enhanced by HDAC1 siRNA or inhibitors. Unlike captopril, pathological stimuli such as Ang II, ET-1, and H_2_O_2_ increased acetylation and ubiquitination levels of Sp1/Sp3, thus suppressing AMPKα1/AMPKα2 transcription. This study identified a molecular mechanism by which captopril improves endothelial function and protects against hypertension.

We previously reported that administration of ACEI significantly improved angiogenesis and exerted protective effects in CTR mice, but failed to rescue the deleterious phenotype resulting from endothelial Sp1/Sp3 deletion. We have identified USP7 as an ACEI-activated deubiquitinating enzyme that translocated into the nucleus binding to Sp1/Sp3, which are deacetylated by HDAC1^15^. In this study, we also demonstrated that acetylation regulated by HDAC1 and ubiquitination played a vital role in captopril-mediated regulation of Sp1/Sp3 and further antihypertensive efficacy. Indeed, USP7 and HDAC1 are known to play important roles in regulating the activity or abundance of numerous genes and proteins. We restricted our investigation to the effect of HDAC1-mediated deacetylation on Sp1/Sp3 binding with USP7, however, the relationships between the HDAC1 and USP7 require further investigation.

## Methods

### Ethical statement

Animal experiments were approved by the Animal Care Committee of Shandong University and were performed in compliance with the Animal Management Rules of the Chinese Ministry of Health. All animal experiments conformed to the guidelines of Directive 2010/63/EU of the European Parliament on the protection of animals used for scientific purposes. All ethical guidelines were adhered to whilst carrying out this study.

### Cell culture and treatments

Human umbilical vein endothelial cells (HUVECs) were isolated from normal human umbilical veins, which were collected from Qilu Hospital of Shandong University. Isolation of the HUVECs had IRB approval and the patients gave written informed consent. HUVECs were cultured in Endothelial Cell Medium (ECM; Sciencell) supplemented with 5% heat-inactivated fetal bovine serum (ScienCell), 1% penicillin/streptomycin (ScienCell), and 1% endothelial cell growth supplement (ECGS; ScienCell). HEK293T cells were obtained from American Type Culture Collection (ATCC, Manassas, VA, USA) and cultured in DMEM/high-glucose media (Gibico, NY, USA) supplemented with 10% fetal bovine serum (Gibico, NY, USA) and antibiotics. The Bovine aortic endothelial cells (BAECs) was obtained from OTWO (Guangzhou, China) and cultured in Medium 199 (Gibico, NY, USA) with 10% fetal bovine serum and antibiotics. All cells were cultured at 37 °C with 5% CO_2_. Cells at passages 3 to 8 were used.

Stimuli were used in this study: (1) Ang II (a final concentration of 1 μmol/L in culture medium, ApexBio, TX, USA, Cat# A1042). (2) ET-1 (a final concentration of 100 nmol/L in culture medium, MCE, NJ, USA, Cat# HY-P0202). (3) H_2_O_2_ (a final concentration of 200 μmol/L in culture medium, Sigma-Aldrich, MO, USA, Cat# 88597). (4) Captopril (a final concentration of 10 μmol/L in culture medium, Sigma-Aldrich, MO, USA, Cat# C4042). (5) des-Arg9-[Leu8] bradykinin (B1 blocker, a final concentration of 1 μmol/L in culture medium, Sigma-Aldrich, MO, USA, Cat# B6929). (6) Fasitibant chloride hydrochloride (B2 blocker, a final concentration of 1 μmol/L in culture medium, MCE, NJ, USA, Cat# HY-106277A). (7) Trichostatin A (TSA, a final concentration of 10 μmol/L in culture medium, Sigma-Aldrich, MO, USA, Cat# T1952). (8) Vorinostat (SAHA, a final concentration of 10 μmol/L in culture medium, Sigma-Aldrich, MO, USA, Cat# SML0061). (9) Mithramycin A (MITA, a final concentration of 200 nmol/L in culture medium, MCE, NJ, USA, Cat# HY-A0122). (10) 3-Methyladenine (3-MA, a final concentration of 5 mmol/L in culture medium, MCE, NJ, USA, Cat# HY-19312). (11) Chloroquine (CQ, a final concentration of 20 μmol/L in culture medium, MCE, NJ, USA, Cat# HY-17589A). (12) MG-132 (a final concentration of 10 μmol/L in culture medium, MCE, NJ, USA, Cat# HY-13259). (13) N-acetyl-L-cysteine (NAC, a final concentration of 300 μmol/L in culture medium, Sigma-Aldrich, MO, USA, Cat# A7250). (13) Des-Arg9-Bradykinin (a final concentration of 1 μmol/L in culture medium, MCE, NJ, USA, Cat# HY-P0298).

### Animals

Mice expressing the Cre recombinase under control of the VE-Cadherin promoter/ enhancer (*VE-CAD-CreER*^*T2*^) were a gift from Prof. Yulong He in Soochow University^41^. Sp1-floxed (*Sp1*^*fl/fl*^) and Sp3-floxed (*Sp3*^*fl/fl*^) mice were obtained from Prof. Sjaak Philipsen (Erasmus University Medical Center, Rotterdam)^14^. As previously described^15^, to ablate Sp1 and Sp3 specifically in the endothelium (Sp1/Sp3^ECKO^), *Sp1*^*fl/fl*^ and *Sp3*^*fl/fl*^ mice were crossbred with *VE-CAD-CreER*^*T2*^ mice to obtain *VE-CAD-CreER*^*T2+*^*/Sp1*^*fl/fl*^*/Sp3*^*fl/fl*^ mice (dKO), which were intraperitoneally injected with tamoxifen (50 mg/kg) for 5 consecutive days. Littermate *VE-CAD-CreER*^*T2-*^*/Sp1*^*fl/fl*^*/Sp3*^*fl/fl*^ mice were treated with the same dose of tamoxifen as controls (CTR). For MITA treatment, C57BL/6J mice were administered MITA (0.4 mg/kg body weight, MCE, NJ, USA) or vehicle via intraperitoneal injection twice a week for 3 weeks as described^42^. For adenoviral injections, CTR or dKO mice received tail vein injections of 100 μL adenoviral vectors that expressed LacZ or constitutively active AMPK (CA-AMPK, 4 × 10^10^ viral particles). For captopril treatment, CTR or dKO mice were administered captopril (10 mg/kg body weight, Sigma-Aldrich, MO, USA) or vehicle via intraperitoneal injection per day for 4 weeks. At the end of the experiments, the mice were terminated by CO_2_ anesthesia.

Mice were housed at 25 °C, 12-h light/dark and were euthanized using an overdose of anesthesia with 1–1.5% isoflurane, followed by exsanguination and tissue removal. Animal experiments were approved by the Animal Care Committee of Shandong University (KYLL-2018-012) and were performed in compliance with the Animal Management Rules of the Chinese Ministry of Health. All animal experiments conformed to the guidelines of Directive 2010/63/EU of the European Parliament on the protection of animals used for scientific purposes.

### Ang II-induced hypertension in mice

Ang II-induced hypertension in mice was performed as described^43^. Male mice at 10 to 12 weeks of age were used in Ang II-induced hypertension model by subcutaneous infusion of Ang II (ApexBio, TX, USA) at a dose of 1000 ng/kg/min or saline using osmotic minipumps (Alzet MODEL 2006; DURECT, CA, USA) for 28 days. Captopril (10 mg/kg body weight, Sigma-Aldrich, MO, USA) or vehicle was used 48 h before minipumps implantation in CTR or dKO mice.

### Histological analysis

Artery samples were fixed in 4% paraformaldehyde solution for paraffin embedding. Frozen tissues were embedded in the O.C.T. compound (Tissue-Tek). Sections were blocked in 5% goat serum for 1 h at room temperature, then incubated with primary antibody at 4 °C overnight. The primary antibodies used in this study was as follows. CD31 (1:100, #ab28364, Abcam; 1:100, #ab9498, Abcam), caveolin-1 (1:50, #3267, Cell Signaling Technology), Sp1 (1:50, #sc−420, Santa Cruz Biotechnology Inc.), Sp3 (1:50, #sc-28305, Santa Cruz Biotechnology Inc.), p-AMPKα (Thr172, 1:100, #50071, Cell Signaling Technology). After washing with phosphate buffered saline, samples were incubated with Alexa Fluor-labeled secondary antibody at room temperature for 1 h including Alexa Fluor 488 (1:200, #ab150077, Abcam; 1:200, #ab150113, Abcam), Alexa Fluor 594 (1:200, #ab150080, Abcam; 1:200, #ab150116, Abcam). Finally, nuclei were stained with 4,6’-diamidino-2-phenylindole (DAPI, #ab104139, Abcam) for 5 min at room temperature, and immunofluorescence was analyzed under a florescent microscope.

### Plasmids and RNA interference

All constructs were confirmed by DNA sequencing. Lipofectamine 2000 reagents (Thermo Scientific, USA) were used for transient transfection of plasmids into HEK293T and HUVECs. Lipofectamine RNAiMAX reagents (Thermo Scientific, USA) were used for transient transfection of siRNA into cells. All siRNA listed in Supplementary Table II were obtained from GenePharma (Shanghai, China).

### Western blot analysis

Cells were lysed in RIPA lysis buffer (#R0010, Solarbio, Beijing, China) with the addition of protease inhibitor (#CW2200, CWBIO, Beijing, China) and phosphatase inhibitor (#CW2383, CWBIO, Beijing, China). Proteins were resolved in 10% SDS-PAGE gels and transferred to PVDF membrane (Millipore, USA). Membranes were blocked for 2 h at room temperature in TBST containing 5% fat-free milk and incubated with primary antibodies at 4 °C overnight. After TBST washing, membranes were incubated with secondary antibody (Proteintech, Wuhan, China) for 1 h at room temperature. After TBST washing, bands were scanned and analyzed by using Amersham Imager 680 (GE, MA, USA). The primary antibodies used in this study were as follows. GAPDH (1:1000, #5174, Cell Signaling Technology), eNOS (1:1000, #32027, Cell Signaling Technology), p-eNOS (Ser1177, 1:1000, #9570, Cell Signaling Technology), AMPKα1 (1:1000, ab32047, Abcam), AMPKα2 (1:1000, ab3760, Abcam), p-AMPKα (Thr172, 1:1000, #50081, Cell Signaling Technology), caveolin-1 (1:1000, #3267, Cell Signaling Technology), p16 (1:1000, #80772, Cell Signaling Technology), p21 (1:1000, #2947, Cell Signaling Technology), ubiquitin (1:1000, #3936, Cell Signaling Technology), Flag-tag (1:1000, #14793, Cell Signaling Technology), HDAC1 (1:1000, #34589, Cell Signaling Technology), p62 (1:1000, #39749, Cell Signaling Technology), Sp1 (1:1000, #07-645, Millipore), Sp3 (1:1000, #ab227856, Abcam), p-ULK1 (Ser555, 1:1000, #5869, Cell Signaling Technology), ULK1 (1:1000, #8054, Cell Signaling Technology), LC3A/B (1:1000, #12741, Cell Signaling Technology), caspase3 (1:1000, #14220, Cell Signaling Technology), cleaved-caspase3 (1:1000, #9661, Cell Signaling Technology), bradykinin B1 receptor (1:1000, #sc-293196, Santa Cruz Biotechnology Inc.).

### Immunoprecipitation (IP)

Cells were lysed in Pierce IP Lysis Buffer (#87788, Thermo Scientific) and protease inhibitor cocktail. Then proteins were quantified using BCA Assay Kit (Solarbio, Beijing, China). For immunoprecipitation analysis, 500 μg of protein from cells were prepared. To preclear the sample, the samples were incubated with 20 μL of Protein A&G magnetic beads (Bimake, Shanghai, China) for 1 h at 4 °C with constant rotation. Simultaneously, 2 μg antibody was incubated with 30 μl magnetic beads at 4 °C overnight with constant rotation. The magnetic beads were then washed 4 times with IP buffer, and immunoprecipitated proteins were eluted from magnetic beads by 1x loading buffer (CWBIO, Beijing, China) and then were incubated at 100 °C for 5 min. The immunoprecipitated proteins were subject to Western blot analysis.

### Blood pressure measurements

Systolic, diastolic, and mean blood pressure of conscious mice was recorded indirectly and non-invasively by using a tail-cuff system (BP-2010E; Softron). After animals were placed in a hop pocket, the sensor was positioned on the base of the tail. Then the hop pockets were kept in a prewarmed box at 37 °C and pressure was measured for 20 min during the daytime (14:00 to 17:00 h) every day. Mice were acclimated to the system for 7 consecutive days before blood pressure measurement. Data were calculated as the mean blood pressure of 3 sequential days, and blood pressure was measured at least 3 times in each animal.

### Measurement of vascular reactivity of mesenteric arteries

After sacrifice of mice, the second-order mesenteric arteries were isolated from adult male mice, mounted on an Automated Multi Wire Myograph System (#630MA, DMT, Aarhus, DK). Briefly, two tungsten wires (40 μm diameter) were carefully inserted into the arterial lumen, and fixed to a force transducer and a micrometer respectively. Mesenteric arteries were bathed in an oxygenated organ chamber containing physiological salt solution (PSS solution, 130 mM NaCl, 4.7 mM KCl, 24.9 mM NaHCO_3_, 1.18 mM KH_2_PO_4_, 1.17 mM MgSO_4_, 1.6 mM CaCl_2_, 0.026 mM EDTA, and 5.5 mM glucose) and set to the baseline circumference. After stabilizing for 30 min, mesenteric artery viability was tested using a potassium-rich solution (60 mM). Endothelium-dependent relaxation (EDR) was induced by acetylcholine (ACh, MCE, NJ, USA, 10^−10^ to 10^−4 ^mol/L) in phenylephrine (Sigma-Aldrich, MO, USA, 10 μmol/L) pre-contracted segments with or without L-nitro-arginine methyl ester (L-NAME, Sigma-Aldrich, MO, USA, 30 min, 100 μmol/L). Cumulative concentration response curves in response to sodium nitroprusside (SNP, Sigma-Aldrich, MO, USA, 10^−10^ to 10^−4 ^mol/L) were used to assess the endothelial-independent relaxation of vessels.

For ex vivo assay, mesenteric arteries from Ang II-induced mice were separately incubated vehicle, captopril (10 μmol/L), lisinopril (10 μmol/L, MCE, NJ, USA, Cat# HY-18206), ARB (valsartan, 10 μmol/L, MCE, NJ, USA, Cat# HY-18204), captopril with B1 blocker (1 μmol/L) and captopril with B2 blocker (1 μmol/L) in PSS solution for 6 h before measurement.

### Primary endothelial cell isolation and culture

Mouse lung endothelial cells (MLECs) were isolated from adult mice as described^44^. Briefly, lungs were harvested, shredded, and digested with 0.1% collagenase in PBS for 45 min. The digest was homogenized by multiple passages through a 20-gauge needle, then filtered through a 70-μm tissue sieve. MLECs were isolated by immunoselection with CD31-conjugated (BD Pharmingen, CA, USA) magnetic beads (Invitrogen, USA). When plated cells reached confluency, a second immuno-isolation was performed with magnetic beads conjugated with intercellular adhesion molecule 2 (BD Pharmingen, CA, USA). Cells from passages 1 to 3 were used.

Mouse aortic endothelial cells (MAECs) were isolated and cultured using the primary explant technique as previously described^45^. Briefly, mice were anaesthetized, the aorta was dissected and immersed immediately in the Kreb’s solution. After fat and connective tissue was carefully cleaned, the vessel was opened longitudinally and cut into small pieces about 1–2 mm^2^ and plated with the intima side down in a fibronectin-coated culture dish. A small amount of culture medium (containing: DMEM/F12, 20% FCS, 25 U/ml heparin, 100 U/ml penicillin, 100 U/ml streptomycin, and 10 ng/ml ECGs) was added into the dish. The explants were placed in an incubator at 37 °C in 5% CO_2_ atmosphere. After 24 h, more medium was added. About 5–7 days, the endothelial cells began to migrate from the aortic segments. When reaching confluence, cells were then subcultured with 0.25% trypsin with 0.02% EDTA. Passage 2 to 5 of endothelial cells were used in this study.

### NO detection (nitrite/nitrate detection)

NO production was determined by detecting the nitrite level using Griess reagent (Jian-Cheng Bioengineering Institute, Nanjing, China) according to the manufacturer’s instructions. The first step converted nitrate to nitrite by using nitrate reductase. The second step used Griess Reagent to convert nitrite to a deep purple azo compound. The amount of the azochromophore accurately reflected nitric oxide amount. The production of NO in both cell lysate and serum was measured at an absorbance of 540 nm. The production of NO in lysate was normalized to protein concentrations. Total protein concentration was measured by the BCA method.

### eNOS activity detection

eNOS activity of endothelial cells was measured by using the Nitric Oxide Synthase Activity Assay Kit (#ab211083, Abcam, Cambridge, UK). In the presence of NADPH, FAD, FMN, (6R)-5,6,7,8-tetrahydrobiopterin, calmodulin and heme, NOS catalyzes a five-electron oxidation of the guanidino nitrogen of L-arginine with molecular oxygen to generate NO and L-citrulline. eNOS activity measurement was based on citrullin conversion. We measured the fluorescence of samples and standard curve on a microplate reader at Ex/Em = 360/450 nm. Protein concentration was determined by the BCA method. eNOS activity was expressed as pmol/mg protein/min.

### Echocardiography

Echocardiography was performed with a Micro-Echocardiography system (Vevo 2100; Visual Sonics). Mice were anesthetized with isoflurane. The fur of mice was removed from the chest with depilatory cream, and animals were placed supine on a 37 °C tempered platform with all legs taped to electrocardiography electrodes for heart rate monitoring. 2D images and M-mode tracings were recorded from the short-axis view at the high papillary muscle level. The following parameters were measured: left ventricular posterior wall thickness, ejection fraction, and fractional shortening.

### RNA-seq analysis

Total RNA was extracted from MLECs of CTR and KO mice using Trizol reagent (Invitrogen, USA) according to manufacturer’s instructions, followed by DNase I treatment to remove DNA contamination. RNA purity was checked using the NanoPhotometer® spectrophotometer (Thermo Fisher, MA, USA). RNA concentration was measured using Qubit® 3.0 Fluorometer (Life Technologies, CA, USA). RNA integrity was assessed using the RNA Nano 6000 Assay Kit of the Bioanalyzer 2100 system (Agilent Technologies, CA, USA). Sequencing libraries were generated using NEB Next® UltraTM RNA Library Prep Kit for Illumina® (NEB, USA) following the manufacturer’s recommendations. The library fragments were purified with AMPure XP system (Beckman Colter, Beverly, USA). The libraries were sequenced on the Illumina novaseq 6000 platform and 150 bp paired-end reads were generated (Anoroad, Beijing, China).

Fastq files were initially subjected to a quality control step using FastQC (v0.10.1), and the reads were then trimmed using Trimmomatic. To accurately quantify human gene expression, we applied a two-step alignment. The filtered reads were first mapped to a combined reference genome from mouse (mm39) using STAR (v2.7.1a). The reads that uniquely aligned to the mouse genome were extracted and converted into fastq format by sambamba. We then aligned the cleaned mouse reads to the mm39 genome for downstream analyses. Differential expression genes (DEGs) were defined using DESeq2 with the adjusted *P* value < 0.05 and absolute log2(fold-change) > 1.5.

### RNA extraction and quantitative RT-PCR

Total RNA was extracted from mouse tissues or cells by using Trizol reagent (Invitrogen, USA) according to the manufacture’s protocol and reverse transcribed by using the HiScript III RT SuperMix for qPCR (Vazyme, Nanjing, China) according to the manufacturer’s protocol. Quantitative PCR was performed with the ChamQ Universal SYBR qPCR Master Mix (Vazyme, Nanjing, China) and a CFX-96 system (Bio-Rad, USA). The relative expression of target genes in various groups was calculated with 2^−ΔΔCt^ methodology. GAPDH was used as housekeeping gene in this study. All primer sequences used for real-time PCR assays are in Supplementary Table I.

### Chromatin immunoprecipitation (ChIP) assay

ChIP assays were performed in HUVECs. In brief, HUVECs with different treatments were incubated with 1% fresh paraformaldehyde at room temperature for 10 min to crosslink the histone/transcription factor complexes with DNA, followed by 0.1% glycine incubation at room temperature for 5 min. The nuclei pellets were sonicated using a sonicator. After centrifugation, the chromatin was immunoprecipitated with antibodies against Sp1 (1:50 dilution, Millipore, #07-645), Sp3 (1:50 dilution, Abcam, ab227856), normal rabbit IgG (CST, #2729), HDAC1 (1:50 dilution, CST, #34589), HDAC2 (1:50 dilution, CST, #57156), HDAC6 (1:50 dilution, CST, #7558), p300 (1:50 dilution, CST, #54062), and CBP (1:50 dilution, CST, #7389) overnight with gentle rotation. The protein/DNA complexes were immunoprecipitated by 30 μl ChIP grade protein G magnetic beads with rotation for 2 h at 4 °C, followed by three washes in low-salt buffer and one wash in high-salt buffer, and elution at 65 °C for 30 min. The eluted protein-DNA complexes were reversed with proteinase K at 65 °C for 2 h. The DNA was purified and then amplified by quantitative real-time PCR with the following primers targeted to the human AMPKα1 promoter (−88/+17), forward primer: 5’-CCGCCTAATCGTTCCAGGAA-3’ and reverse primer: 5’-GGGGCTGCCAGGAGAATC-3’; human AMPKα2 promoter (−204/−94), forward primer: 5’-TGTCGCTGCTTCGGGTTC-3’ and reverse primer: 5’-CAGGTGGGAAGCAACGGG-3’. The ChIP-qPCR data were reported as percentage of input, which can be calculated by the formula:

$$\%\, {{{\mbox{of input}}}}=2^{\wedge}{(({{{{{\rm{Ct}}}}}}({{{{{\rm{input}}}}}})-\log 2({{{{{\rm{dilution\; factor}}}}}}))-{{{{{\rm{Ct}}}}}}({{{{{\rm{ChIP}}}}}}))} * 100\%.$$

The input sample used was 10% of the DNA amount, and thus the dilution factor is 10.

### Luciferase reporter assay

Luciferase reporter transfection and dual-luciferase assay were performed using the Promega Dual-Luciferase® Reporter Assay System. In brief, HUVECs or 293T cells were seeded in 24-well plates and transfected by using Lipofectamine 2000 (Invitrogen, CA, USA) with 100 ng report vector (carrying firefly luciferase, Promega, WI, USA) inserted with indicated target sequences; empty vector was the control. PRL-TK vector (carrying renilla luciferase, Promega, WI, USA) was co-transfected as an internal control. At 48 h after transfection, cells were lysed by using passive lysis buffer (Promega, WI, USA) and subjected to luciferase assay according to the manufacturer’s protocol.

### Flow cytometry

Cell apoptosis after Ang II (MCE, NJ, USA) stimuli was determined by using the FITC Annexin V Apoptosis Detection Kit I (BD Biosciences, CA, USA). Cells were harvested and resuspended in 100 µL of Annexin V binding buffer containing 5 µl propidium iodide (PI) and 5 µl Annexin V-FITC for 15 min at room temperature in the dark. An amount of 400 μl Annexin V binding buffer was added to each tube. A dot plot was set up to detect size (forward scatter; FSC) and granularity (side scatter; SSC) using linear scale. Cellular debris were excluded by setting exclusion gates based on FSC and SSC. An untreated sample was run to adjust the voltage and gain for the FITC and PI detectors so that all cells can be detected in the bottom left quadrant. A treated sample stained with FITC alone was run to adjust the voltage and gain for the FITC detector so that the dead cells appear in the bottom right quadrant. A treated sample stained with PI alone was run to adjust the voltage and gain for the PI detector so that the dead cells appear in the top left quadrant. A treated sample stained with FITC and PI was run to adjust the compensation so that the live cells appear in the bottom left, the apoptotic cells appear in the bottom right, and the necrotic cells appear in the top right quadrants. Then each sample was run for at least 20,000 events. Flow cytometry was performed by using a FACSCalibur flow cytometer (BD Biosciences, CA, USA) and analyzed by FlowJo (version version 10.6.0).

### Human mesenteric artery samples

Human mesenteric arteries were obtained from 10 patients (4 male and 6 female) in the Department of Bariatric and Metabolic Surgery, General Surgery, Qilu Hospital, Shandong University. Five patients with hypertension with an average of 29 were included in this study. 5 patients without hypertension with an average of 27 were included in this study. All patients gave written informed consent to participate in the study. All procedures involving human samples were approved by the Ethical Committee of Qilu Hospital of Shandong University. All relevant ethical regulations were followed in this study.

### Statistics

Data are expressed as means ± SEM, and results were analyzed by using R (for RNA sequencing) and GraphPad Prism 8.0 (GraphPad Software, San Diego, CA). Normality assumption of the data distribution was assessed with the Kolmogorov–Smirnov test. For normally distribution, Student *t* test was used to compare 2 groups. Differences between multiple groups with one variable were determined by one-way ANOVA followed by Bonferroni’s post hoc test. To compare multiple groups with more than one variable, two-way ANOVA followed by Bonferroni’s post hoc test was used. Statistical significance was set at *P* < 0.05.

### Reporting summary

Further information on research design is available in the Nature Portfolio Reporting Summary linked to this article.

### Supplementary information

Supplementary Information

Reporting Summary

### Source data

Source Data

## Data Availability

The RNA-seq data generated in this study have been deposited in the NCBI Gene Expression Omnibus (GEO) database under accession code GSE206586. All other data generated or analyzed during this study are included in this published paper (and its Supplementary Information files). Additional data related to this paper are available from the corresponding author on request. Source data are provided with this paper.
