# Supplementary material for: Endothelial Sp1/Sp3 are essential to the effect of captopril on blood pressure in male mice
Source: Nat Commun. 2023 Sep 21;14:5891. doi: 10.1038/s41467-023-41567-1 (PMC10514286; doi:10.1038/s41467-023-41567-1)
Supplement: Supplementary file 1 — Supplementary Information [file 41467_2023_41567_MOESM1_ESM.pdf]

Supplemental Figures and Figure legends

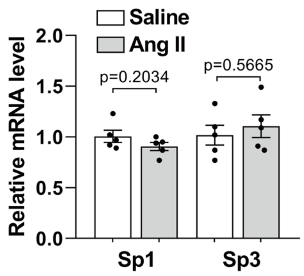

Fig. S1. mRNA level of Sp1 and Sp3 in MAECs.

A, qPCR analysis of of *Sp1*/*Sp3* in mouse aortic endothelial cells (MAECs) isolated from Saline or AngII-treated mice. n = 5. Data are presented as mean ± SEM. Two-tailed Student unpaired t test for **A**.

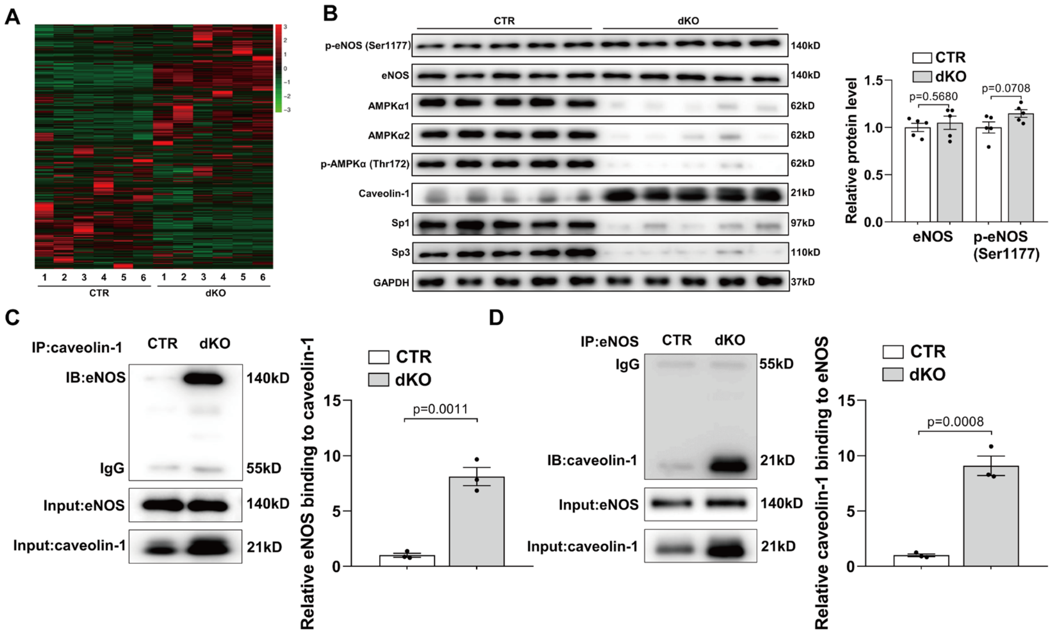

Fig. S2. Analysis of MLECs from CTR and dKO mice.

A, Heatmap represents genes with >1.5-fold upregulation or >1.5-fold downregulation in MLECs from dKO mice compared with CTR. n = 6. **B**, WB of p-eNOS (Ser1177), eNOS, Sp1, Sp3, AMPKα1, AMPKα2, p-AMPKα (Thr172), caveolin-1 in MAECs from CTR and dKO mice. Quantitative analysis of eNOS and p-eNOS (Ser1177) (right). n = 5. **C**, Co-immunoprecipitation (CoIP) analysis of eNOS immunoprecipitated with

anti-caveolin-1 antibody in MLECs from CTR and dKO mice. **D**, CoIP analysis of caveolin-1 immunoprecipitated with anti-eNOS antibody in mouse lung endothelial cells (MLECs) from CTR and dKO mice. n = 3. Data are presented as mean ± SEM. Two-tailed Student unpaired t test for **C** and **D**.

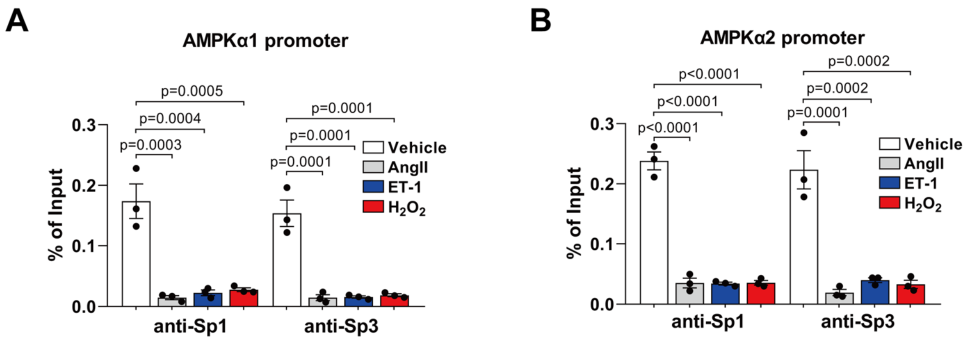

Fig. S3. Ang II/ET-1/H_2O_2 inhibited Sp1/Sp3 binding to AMPKα1/AMPKα2 promoter.

A and **B**, Chromatin immunoprecipitation (ChIP) assay showing the binding of Sp1 or Sp3 to the (**A**) AMPKα1 and (**B**) AMPKα2 promoter in HUVECs with different treatments. n = 3. Data are presented as mean ± SEM. One-way ANOVA followed by Bonferroni post hoc analysis for **A** and **B**.

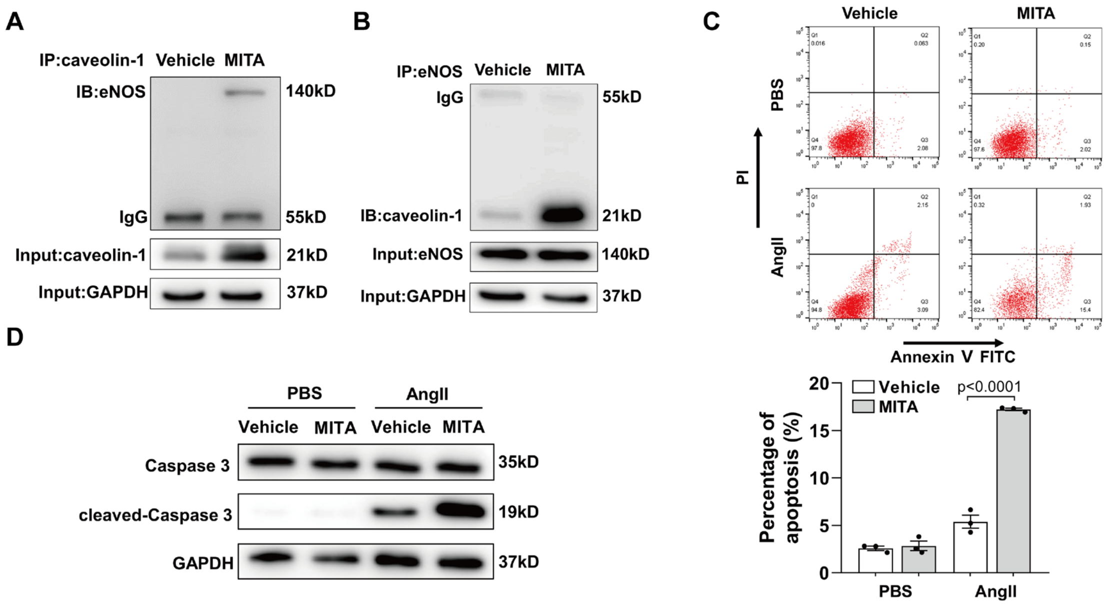

Fig. S4. CoIP analysis of eNOS and caveolin-1 and apoptosis analysis in MLECs from vehicle and MITA treated mice.

A and **B**, CoIP assay of (**A**) eNOS immunoprecipitated with anti-caveolin-1 antibody and (**B**) caveolin-1 immunoprecipitated with anti-eNOS antibody in MLECs from vehicle and MITA treated mice. **C**, MLECs from vehicle and MITA treated mice with different treatments were stained with fluorescein isothiocyanate (FITC)-conjugated annexin V and propidium iodide (PI) and analyzed by flow cytometry. n = 3. **D**, WB analysis of caspase 3 and cleaved-caspase 3 protein levels in MLECs from vehicle and MITA treated mice with different treatments. Data are presented as mean ± SEM. One-way ANOVA followed by Bonferroni post hoc analysis for **C**.

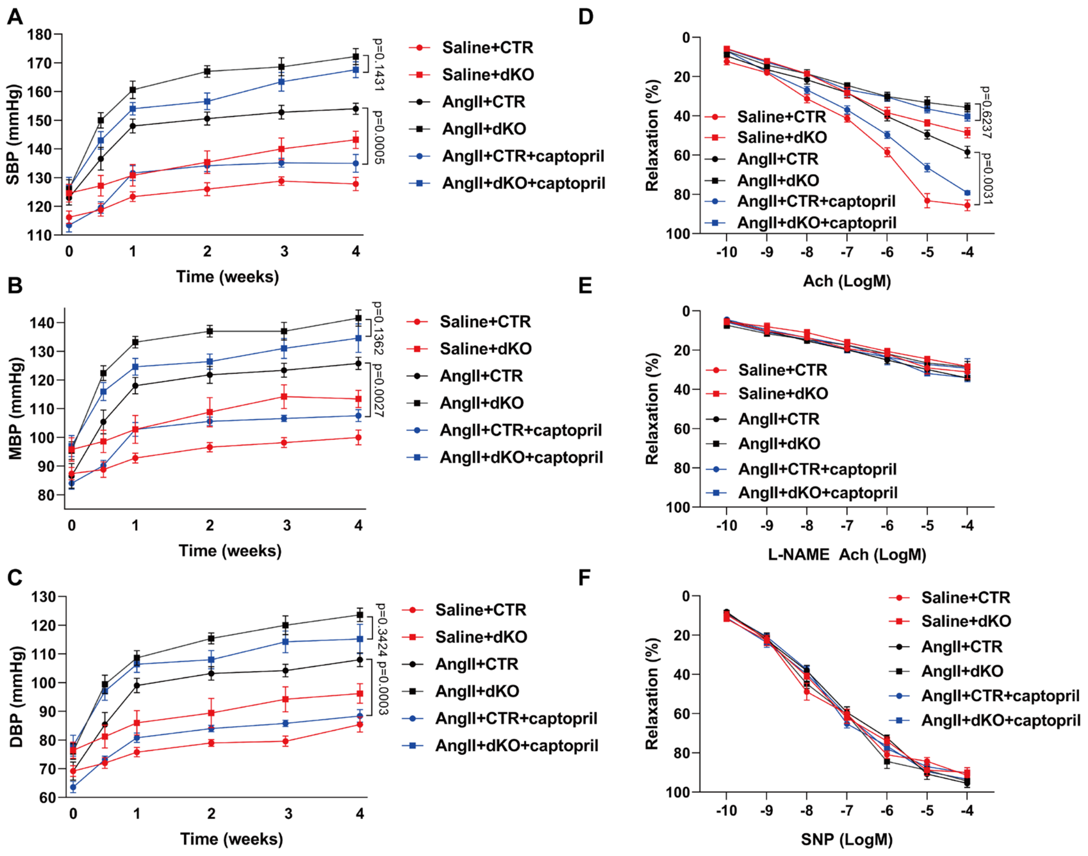

Fig. S5. Endothelial Sp1 and Sp3 are responsible for captopril-mediated actions in Ang II-induced hypertensive mice

A to **C**, Systolic blood pressure (SBP), mean blood pressure (MBP) and diastolic blood pressure (DBP) in different groups of mice. n = 5. **D** to **F**, Vascular reactivity of mesenteric arteries in different groups of mice to Ach with or without L-NAME pretreatment (10^{-4} mol/L, 30 min) and SNP. n = 5. Data are presented as mean ± SEM. Two-way ANOVA followed by Bonferroni post hoc test for **A** to **E**.

.

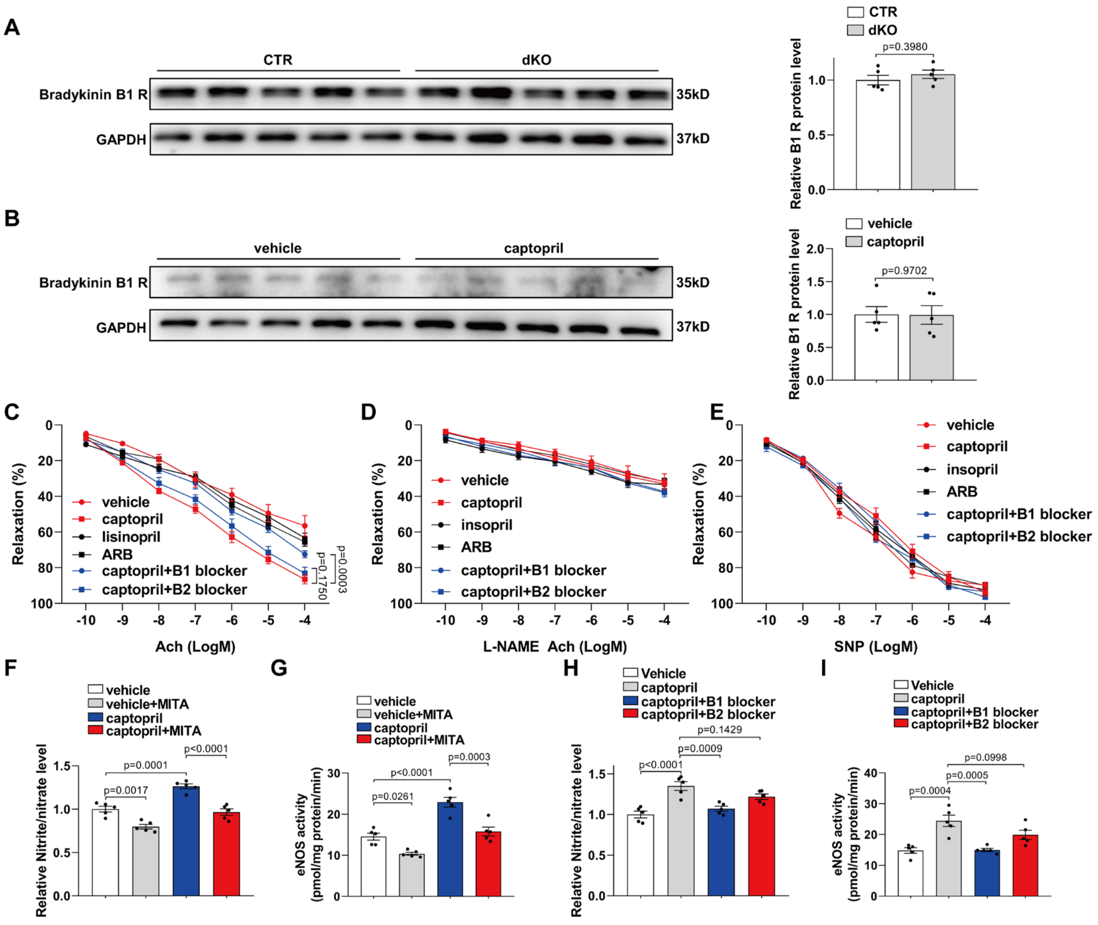

Fig. S6. Captopril directly activates bradykinin B1 receptor.

A, WB analysis of bradykinin B1 receptor in MLECs from CTR and dKO mice. n = 5. **B**, WB analysis of bradykinin B1 receptor in HUVECs treated with vehicle or captopril. n = 5. **C** to **E**, Mesenteric arteries from Ang II-induced hypertensive mice were respectively incubated in vehicle, captopril (10 μmol/L), lisinopril (10 μmol/L), ARB (valsartan, 10 μmol/L), captopril with B1 blocker (1 μmol/L) and captopril with B2 blocker (1 μmol/L) for 6 h before measurement. Then, vascular reactivity of mesenteric resistance arteries to Ach with or without L-NAME pretreatment (10^{-4} mol/L, 30 min) and SNP. n = 5. **F** and **G**, No production (**F**) and eNOS activity (**G**) of BAECs with different treatments. n = 5. **H** and **I**, No production (**H**) and eNOS activity (**I**) in BAECs with different treatments. n = 5. Data are presented as mean ± SEM. Two-tailed Student unpaired t test for **A** and **B**. One-way ANOVA followed by Bonferroni post hoc analysis for **F** to **I**. Two-way ANOVA followed by Bonferroni post hoc test for **C**.

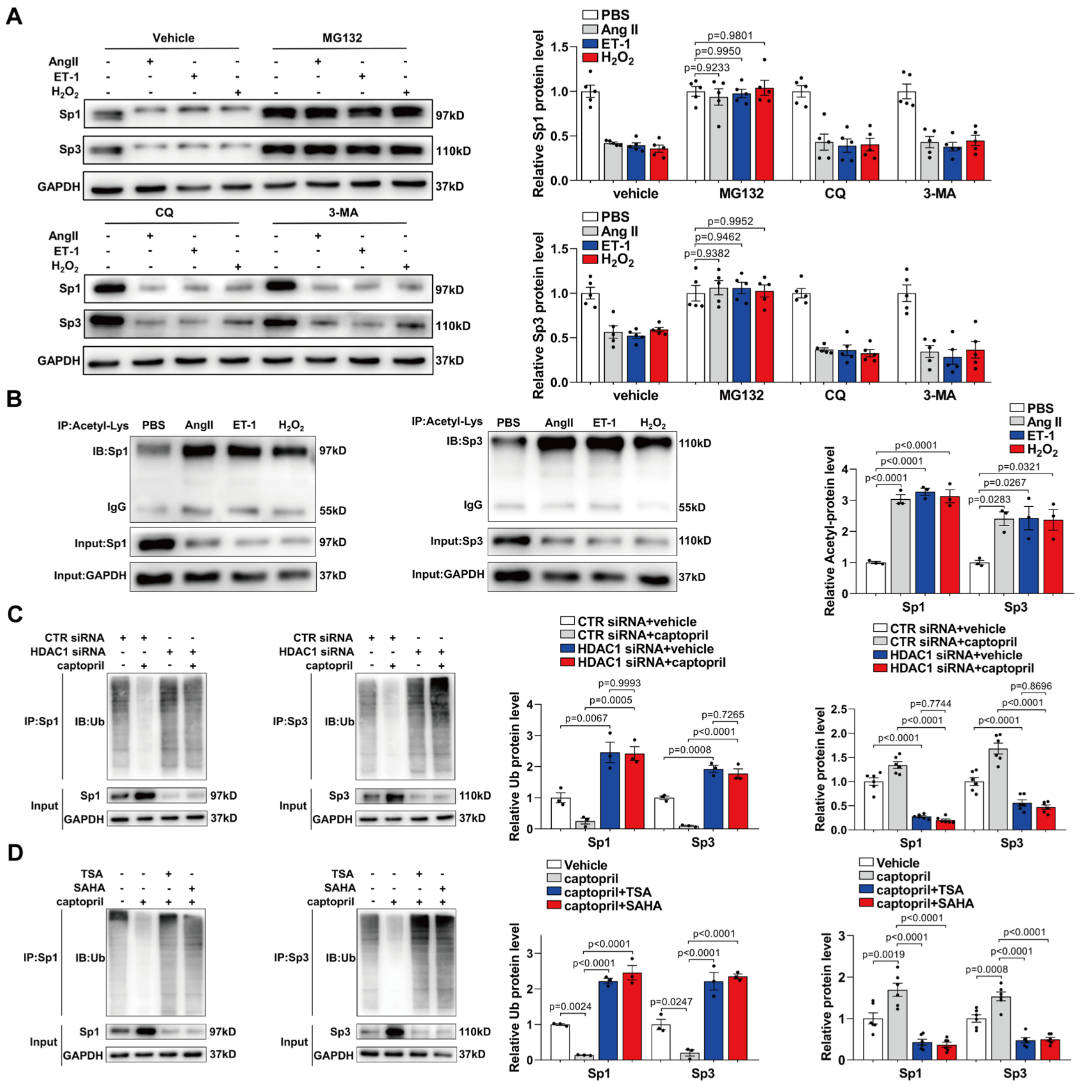
A
Vehicle
MG132
CQ
3-MA
AngII
ET-1
H_2O_2
Sp1
Sp3
GAPDH
97kD
110kD
37kD
PBS
Ang II
ET-1
H_2O_2
Relative Sp1 protein level
Relative Sp3 protein level
vehicle
MG132
CQ
3-MA
p=0.9801
p=0.9950
p=0.9233
p=0.9952
p=0.9462
p=0.9382
B
IP:Acetyl-Lys
PBS
AngII
ET-1
H_2O_2
IB:Sp1
IB:Sp3
IgG
Input:Sp1
Input:Sp3
Input:GAPDH
97kD
55kD
110kD
37kD
Relative Acetyl-protein level
p<0.0001
p=0.0321
p=0.0267
p=0.0283
Sp1
Sp3
C
CTR siRNA
HDAC1 siRNA
captopril
IP:Sp1
IP:Sp3
IB:Ub
Input
Sp1
Sp3
GAPDH
CTR siRNA+vehicle
CTR siRNA+captopril
HDAC1 siRNA+vehicle
HDAC1 siRNA+captopril
Relative Ub protein level
Relative protein level
p=0.9993
p=0.0005
p=0.0067
p=0.7265
p=0.0001
p=0.0008
p=0.8696
p<0.0001
p=0.7744
p<0.0001
D
TSA
SAHA
captopril
Vehicle
captopril
captopril+TSA
captopril+SAHA
p<0.0001
p=0.0024
p=0.0247
p=0.0019
p=0.0008

Fig. S7. Ubiquitination and HDAC1-mediated deacetylation regulate the expression of Sp1/Sp3.

A, WB analysis of Sp1 and Sp3 in HUVECs with different treatments. Quantified western blot analysis of Sp1 or Sp3 protein level (right). n = 5. **B**, CoIP assays of acetylation levels of Sp1 or Sp3 in HUVECs with different treatments. Quantified western blot analysis of acetyl-Sp1 or acetyl-Sp3 protein level (right). n = 3. **C**, CoIP assays of Sp1 or Sp3 ubiquitination in HUVECs transfected with CTR or HDAC1 siRNA treated with captopril. Quantified western blot analysis of ubiquitin, Sp1 or Sp3 protein level (right). n = 3. **D**, Co-IP assay of Sp1 or Sp3 ubiquitination in HUVECs with HDAC1 inhibitor TSA or SAHA. Quantified western blot analysis of ubiquitin, Sp1 or Sp3 protein level (right). n = 3. Data are presented as mean ± SEM. One-way ANOVA followed by Bonferroni post hoc analysis for **A** and **B**. Two-way ANOVA followed by Bonferroni post hoc test for **C** and **D**.

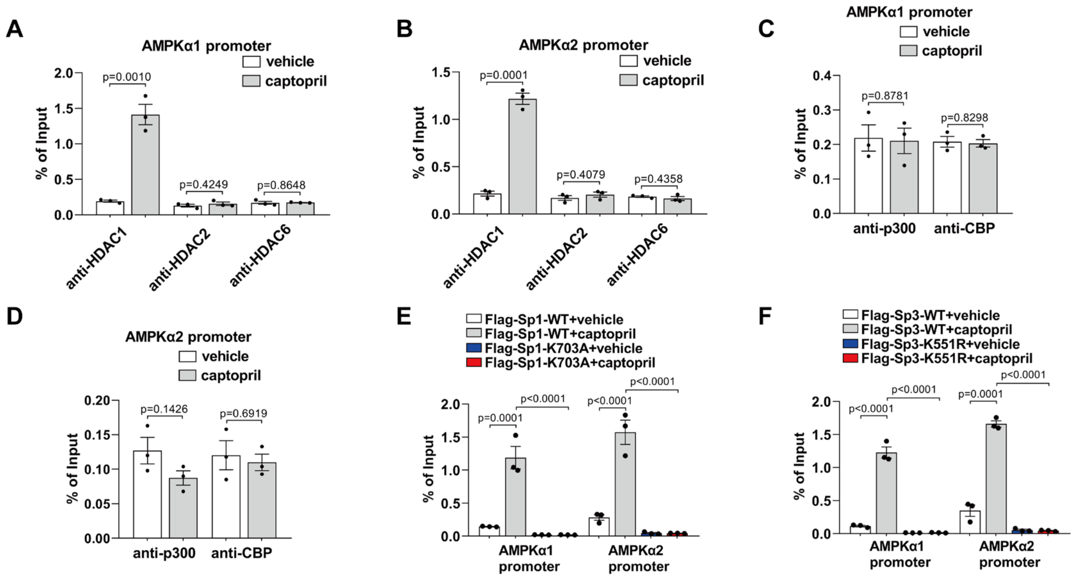

Fig. S8. ChIP analysis in HUVECs treated with captopril.

A, ChIP assay showing the binding of HDAC1, HDAC2, and HDAC6 to the AMPKα1 promoter in HUVECs treated with captopril. n = 3. **B**, ChIP assay showing the binding of HDAC1, HDAC2, and HDAC6 to the AMPKα2 promoter in HUVECs treated with captopril. n = 3. **C**, ChIP assay showing the binding of p300 and CBP to the AMPKα1 promoter in HUVECs treated with captopril. n = 3. **D**, ChIP assay showing the binding of p300 and CBP to the AMPKα2 promoter in HUVECs treated with captopril. n = 3. **E**, ChIP assay showing the binding of Sp1 to the AMPKα1/ AMPKα2 promoter in HUVECs transfected with different plasmids using anti-Flag antibody. n = 3. **F**, ChIP assay showing the binding of Sp3 to the AMPKα1/ AMPKα2 promoter in HUVECs transfected with different plasmids using anti-Flag antibody. n = 3. Data are presented as mean ± SEM. Two-way ANOVA followed by Bonferroni post hoc analysis for **E** and **F**. Two-tailed Student unpaired t test for **A** to **D**.

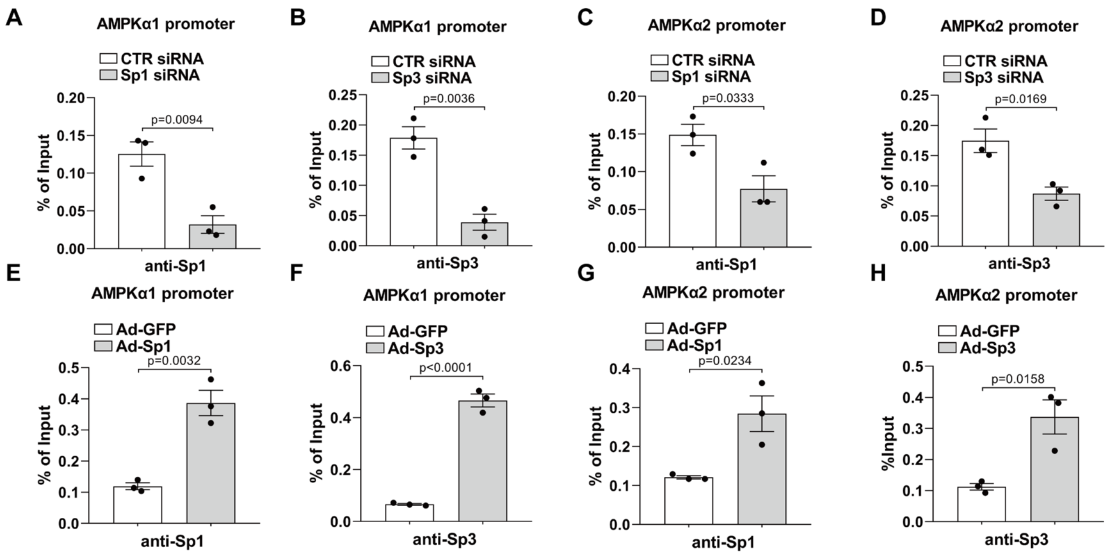

Fig. S9. ChIP analysis in Sp1/Sp3 knockdown- or overexpression-HUVECs

A, ChIP assay showing the binding of Sp1 to the AMPKα1 promoter in HUVECs transfected with CTR siRNA or Sp1 siRNA. n = 3. **B**, ChIP assay showing the binding of Sp3 to the AMPKα1 promoter in HUVECs transfected with CTR siRNA or Sp3 siRNA. n = 3. **C**, ChIP assay showing the binding of Sp1 to the AMPKα2 promoter in HUVECs transfected with CTR siRNA or Sp1 siRNA. n = 3. **D**, ChIP assay showing the binding of Sp3 to the AMPKα2 promoter in HUVECs transfected with CTR siRNA or Sp3 siRNA. n = 3. **E**, ChIP assay showing the binding of Sp1 to the AMPKα1 promoter in HUVECs infected with Ad-GFP or Ad-Sp1. n = 3. **F**, ChIP assay showing the binding of Sp3 to the AMPKα1 promoter in HUVECs infected with Ad-GFP or Ad-Sp3. n = 3. **G**, ChIP assay showing the binding of Sp1 to the AMPKα2 promoter in HUVECs infected with Ad-GFP or Ad-Sp1. n = 3. **H**, ChIP assay showing the binding of Sp3 to the AMPKα2 promoter in HUVECs infected with Ad-GFP or Ad-Sp3. n = 3. Data are presented as mean ± SEM. Two-tailed Student unpaired t test for **A** to **H**.

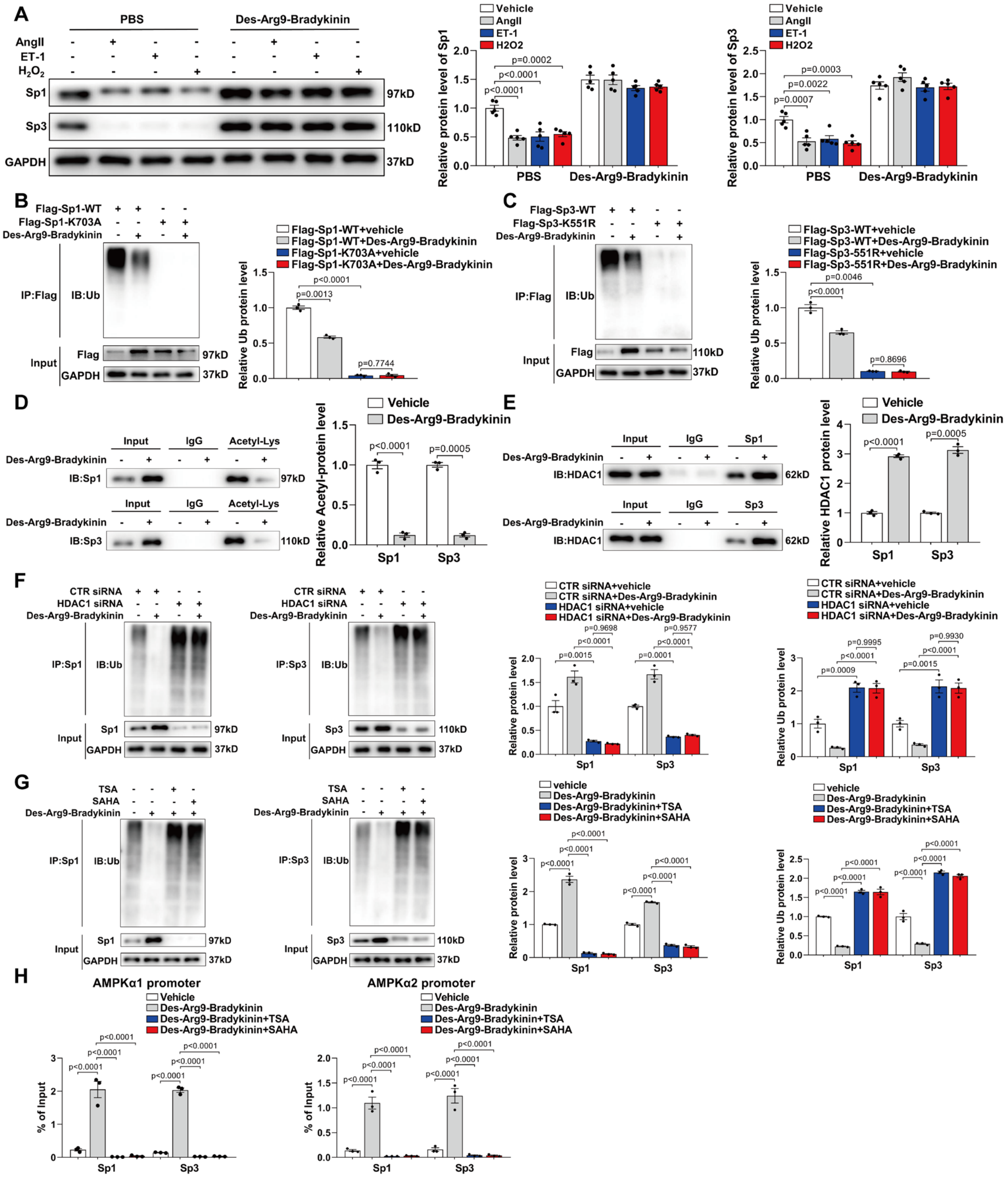
A
PBS
Des-Arg9-Bradykinin
AngII
ET-1
H2O2
Sp1
Sp3
GAPDH
97kD
110kD
37kD
Vehicle
AngII
ET-1
H2O2
Relative protein level of Sp1
Relative protein level of Sp3
p<0.0001
p<0.0001
p=0.0002
p=0.0007
p=0.0022
p=0.0003
B
Flag-Sp1-WT
Flag-Sp1-K703A
Des-Arg9-Bradykinin
IP:Flag
IB:Ub
Input
Flag
GAPDH
Flag-Sp1-WT+vehicle
Flag-Sp1-WT+Des-Arg9-Bradykinin
Flag-Sp1-K703A+vehicle
Flag-Sp1-K703A+Des-Arg9-Bradykinin
Relative Ub protein level
p<0.0001
p=0.0013
p=0.7744
C
Flag-Sp3-WT
Flag-Sp3-K551R
Flag-Sp3-WT+vehicle
Flag-Sp3-WT+Des-Arg9-Bradykinin
Flag-Sp3-551R+vehicle
Flag-Sp3-551R+Des-Arg9-Bradykinin
p=0.0046
p<0.0001
p=0.8696
D
Input
IgG
Acetyl-Lys
IB:Sp1
IB:Sp3
Vehicle
Des-Arg9-Bradykinin
Relative Acetyl-protein level
p<0.0001
p=0.0005
E
IB:HDAC1
62kD
Relative HDAC1 protein level
p<0.0001
p=0.0005
F
CTR siRNA
HDAC1 siRNA
IP:Sp1
IP:Sp3
CTR siRNA+vehicle
CTR siRNA+Des-Arg9-Bradykinin
HDAC1 siRNA+vehicle
HDAC1 siRNA+Des-Arg9-Bradykinin
Relative protein level
Relative Ub protein level
p=0.9698
p<0.0001
p=0.0015
p=0.9577
p=0.0001
p=0.9995
p=0.0009
p=0.9930
G
TSA
SAHA
vehicle
Des-Arg9-Bradykinin
Des-Arg9-Bradykinin+TSA
Des-Arg9-Bradykinin+SAHA
H
AMPKα1 promoter
AMPKα2 promoter
Vehicle
% of Input

Fig. S10. Des-Arg9-Bradykinin regulates Sp1/Sp3 via ubiquitination and HDAC1-mediated deacetylation.

A, WB analysis of Sp1 and Sp3 protein levels in HUVECs with different treatments. Quantified analysis of Sp1 or Sp3 protein level (right). n = 5. **B**, CoIP assay of Sp1 ubiquitination in HUVECs transfected with Flag-Sp1-WT or Flag-Sp1-K703A with different treatments. Quantified analysis of ubiquitin protein level (right). n = 3. **C**, CoIP analysis of Sp3 ubiquitination in HUVECs transfected with Flag-Sp3-WT or Flag-Sp3-K551R with different treatments. Quantified of ubiquitin protein level (right). n = 3. **D**, CoIP assay of acetylation levels of Sp1 or Sp3 in HUVECs treated with Des-Arg9-Bradykinin. Quantified analysis of acetyl-protein level (right). n = 3. **E**, CoIP assay of HDAC1 in HUVECs treated with Des-Arg9-Bradykinin. Quantified analysis of HDAC1 protein level (right). n = 3. **F**, CoIP assay of Sp1 or Sp3 ubiquitination in HUVECs transfected with CTR or HDAC1 siRNA treated with Des-Arg9-Bradykinin. Quantified analysis of ubiquitin, Sp1 or Sp3 protein level (right). n = 3. **G**, CoIP assay of Sp1 or Sp3 ubiquitination in HUVECs with HDAC1 inhibitor TSA or SAHA. Quantified analysis of ubiquitin, Sp1 or Sp3 protein level (right). n = 3. **H**, ChIP assay showing the binding of Sp1 or Sp3 to the AMPKα1 and AMPKα2 promoter in HUVECs with different treatments. n = 3. Data are presented as mean ± SEM. Two-tailed Student unpaired t test for **D** and **E**. One-way ANOVA followed by Bonferroni post hoc analysis for **H**. Two-way ANOVA followed by Bonferroni post hoc test for **A**, **B**, **C**, **F** and **G**.

Table S1. Primer sequences used in this study.

Gene name	Primer sequences for ChIP and qPCR (5'-3')
Human AMPKα1	Forward: AGAAGCAGAAACACGACGGG
	Reverse: CTTCACTTTGCCGAAGGTGC
Human AMPKα2	Forward: CGAAGATGGCTGAGAAGCAGA
	Reverse: TCTCCAATCTTCACTTTGCCGA
Human AMPKα1 (ChIP)	Forward: CCGCCTAATCGTTCCAGGAA
	Reverse: GGGGCTGCCAGGAGAATC
Human AMPKα2 (ChIP)	Forward: TGTCGCTGCTTCGGGTTC
	Reverse: CAGGTGGGAAGCAACGGG
Human Sp1	Forward: TGGCAGCAGTACCAATGGC
	Reverse: CCAGGTAGTCCTGTCAGAACTT
Human Sp3	Forward: GCGACAGGTGATTTGGCTTCT
	Reverse: TACTGCCCACTTGAAGTAGCA
Human β-actin	Forward: GGAAATCGTGCGTGACATTAA
	Reverse: AGGAAGGAAGGCTGGAAGAG
Mouse AMPKα1	Forward: GGGTGAAGATCGGCCACTAC
	Reverse: CTCCGAATCTTCTGCCGGTT
Mouse AMPKα2	Forward: GGCAAAGTGAAGACTACCAGG
	Reverse: CTTCAACCCGCCCATGTTTG
Mouse β-actin	Forward: CCACACCCGCCACCAGTTCG

	Reverse: TACAGCCCGGGGAGCATCGT
Mouse Sp1	Forward: AGGGTCCGAGTCAGTCAGG
	Reverse: CTCGCTGCCATTGGTACTGTT
Mouse Sp3	Forward: AATCAAACCTTACTCGCCTCTG
	Reverse: GCACATTAGCGACTACTTGAGTT

Table S2. siRNA sequences used in this study.

Gene name	Primer sequences for siRNA (5'-3')
Human Sp1	Sense: GUGCAAACCAACAGAUUAUTT
	Antisense: AUAAUCUGUUGGUUUGCACTT
Human Sp3	Sense: GCAGUCAAAUUCAGAUCAUTT
	Antisense: AUGAUCUGAAUUUGACUGCTT
Human AMPKα	Sense: GAGGAUGCCUCAGGAAAUA
	Antisense: AAAGCGUCUGGAAAAGUCG
Human HDAC1	Sense: GCUCCUCUGACAAACGAAUTT
	Antisense: AUUCGUUUGUCAGAGGAGCTT
Negative control	Sense: UUCUCCGAACGUGUCACGUTT
	Antisense: ACGUGACACGUUCGGAGAATT

Reference

1 Monvoisin, A. *et al.* VE-cadherin-CreERT2 transgenic mouse: a model for inducible recombination in the endothelium. *Dev Dyn* **235**, 3413-3422, doi:10.1002/dvdy.20982 (2006).

2 Meinders, M. *et al.* Sp1/Sp3 transcription factors regulate hallmarks of megakaryocyte maturation and platelet formation and function. *Blood* **125**, 1957-1967, doi:10.1182/blood-2014-08-593343 (2015).

3 Lu, H. *et al.* Angiotensin-converting enzyme inhibitor promotes angiogenesis through Sp1/Sp3-mediated inhibition of notch signaling in male mice. *Nature communications* **14**, 731, doi:10.1038/s41467-023-36409-z (2023).

4 Jia, Z. *et al.* Combined treatment of pancreatic cancer with mithramycin A and tolfenamic acid promotes Sp1 degradation and synergistic antitumor activity. *Cancer research* **70**, 1111-1119, doi:10.1158/0008-5472.Can-09-3282 (2010).

5 Guo, J. *et al.* Endothelial SIRT6 Is Vital to Prevent Hypertension and Associated Cardiorenal Injury Through Targeting Nkx3.2-GATA5 Signaling. *Circ Res* **124**, 1448-1461, doi:10.1161/circresaha.118.314032 (2019).

6 Wang, J., Niu, N., Xu, S. & Jin, Z. G. A simple protocol for isolating mouse lung endothelial cells. *Scientific reports* **9**, 1458, doi:10.1038/s41598-018-37130-4 (2019).

7 Yang, H. *et al.* Decrease of intracellular chloride concentration promotes endothelial cell inflammation by activating nuclear factor-κB pathway. *Hypertension* **60**, 1287-1293, doi:10.1161/hypertensionaha.112.198648 (2012).
