# Supplementary material for: Endothelial Sp1/Sp3 are essential to the effect of captopril on blood pressure in male mice
Source: Nat Commun. 2023 Sep 21;14:5891. doi: 10.1038/s41467-023-41567-1 (PMC10514286; doi:10.1038/s41467-023-41567-1)
Supplement: Supplementary file 2 — Reporting Summary [file 41467_2023_41567_MOESM2_ESM.pdf]

nature portfolio

Corresponding author(s): Wencheng Zhang

Last updated by author(s): Aug 21, 2023

Reporting Summary

Nature Portfolio wishes to improve the reproducibility of the work that we publish. This form provides structure for consistency and transparency in reporting. For further information on Nature Portfolio policies, see our Editorial Policies and the Editorial Policy Checklist.

Statistics

For all statistical analyses, confirm that the following items are present in the figure legend, table legend, main text, or Methods section.

n/a	Confirmed	
☐	☒	The exact sample size (*n*) for each experimental group/condition, given as a discrete number and unit of measurement
☐	☒	A statement on whether measurements were taken from distinct samples or whether the same sample was measured repeatedly
☐	☒	The statistical test(s) used AND whether they are one- or two-sided<br>*Only common tests should be described solely by name; describe more complex techniques in the Methods section.*
☒	☐	A description of all covariates tested
☐	☒	A description of any assumptions or corrections, such as tests of normality and adjustment for multiple comparisons
☐	☒	A full description of the statistical parameters including central tendency (e.g. means) or other basic estimates (e.g. regression coefficient) AND variation (e.g. standard deviation) or associated estimates of uncertainty (e.g. confidence intervals)
☐	☒	For null hypothesis testing, the test statistic (e.g. *F*, *t*, *r*) with confidence intervals, effect sizes, degrees of freedom and *P* value noted<br>*Give P values as exact values whenever suitable.*
☒	☐	For Bayesian analysis, information on the choice of priors and Markov chain Monte Carlo settings
☒	☐	For hierarchical and complex designs, identification of the appropriate level for tests and full reporting of outcomes
☒	☐	Estimates of effect sizes (e.g. Cohen's *d*, Pearson's *r*), indicating how they were calculated

Our web collection on statistics for biologists contains articles on many of the points above.

Software and code

Policy information about availability of computer code

Data collection	The softwares to collect the data: Amersham Imager 680; LightCycler 480 II;Pannoramic Scan; Leica H-700FA; Zeiss ZEN 2012
Data analysis	The softwares to analyse the data: SPSS23.0; Graphpad Prism 8; Image J v18.0; Image Pro Plus 6.0; FlowJo V10

For manuscripts utilizing custom algorithms or software that are central to the research but not yet described in published literature, software must be made available to editors and reviewers. We strongly encourage code deposition in a community repository (e.g. GitHub). See the Nature Portfolio guidelines for submitting code & software for further information.

Data

Policy information about availability of data

All manuscripts must include a data availability statement. This statement should provide the following information, where applicable:

- Accession codes, unique identifiers, or web links for publicly available datasets
- A description of any restrictions on data availability
- For clinical datasets or third party data, please ensure that the statement adheres to our policy

Gene expression RNA-seq data has been deposited at the NCBI Gene Expression Omnibus (GEO). The accession number is GSE206586 [https://www.ncbi.nlm.nih.gov/geo/query/acc.cgi?acc=GSE206586]. Data supporting the findings of this study are available within the article and its Supplementary Information files. All relevant data are available from the authors on reasonable request.

Human research participants

Policy information about studies involving human research participants and Sex and Gender in Research.

Reporting on sex and gender	The sex was not considered in the analysis.
Population characteristics	Human mesenteric arteries were obtained from 10 patients (4 male and 6 female) in the Department of Bariatric and Metabolic Surgery, General Surgery, Qilu Hospital, Shandong University. Five patients with hypertension with an average of 29 were included in this study. 5 patients without hypertension with an average of 27 were included in this study.
Recruitment	These patients were recruited while in hospital undergoing bariatric surgery. The sex and age was not considered in the analysis. There were no obvious study selection biases that would have influenced the results except for the sample size, thereby increasing variability.
Ethics oversight	Ethics Committee of Qilu Hospital of Shandong University

Note that full information on the approval of the study protocol must also be provided in the manuscript.

Field-specific reporting

Please select the one below that is the best fit for your research. If you are not sure, read the appropriate sections before making your selection.

- [x] Life sciences
- [ ] Behavioural & social sciences
- [ ] Ecological, evolutionary & environmental sciences

For a reference copy of the document with all sections, see nature.com/documents/nr-reporting-summary-flat.pdf

Life sciences study design

All studies must disclose on these points even when the disclosure is negative.

Sample size	We used standard sample sizes reported in the literature previously in mouse studies. The sample size of animal experiments according to similar studies in the field was chose and at least 5 mice per group was used. The number of the independent experiments for cell and biological experiments was indicated in each figure legend.
Data exclusions	Except where experiments failed because of technical issues, no data was excluded.
Replication	All in vivo and in vitro experiments were highly reproducible and were independently repeated at least 3 times. All experiments were performed independently multiple times using biologically independent replicates. All attempts at replication were successful.
Randomization	Mice were randomly assigned to groups. Cells were grown under the same conditions and randomly allocated into different groups without any bias.
Blinding	The investigators were blinded to group allocation during data collection and analysis. We collected and analyzed the compared samples under the same conditions.

Reporting for specific materials, systems and methods

We require information from authors about some types of materials, experimental systems and methods used in many studies. Here, indicate whether each material, system or method listed is relevant to your study. If you are not sure if a list item applies to your research, read the appropriate section before selecting a response.

Materials & experimental systems

n/a	Involved in the study	
[ ]	[x]	Antibodies
[ ]	[x]	Eukaryotic cell lines
[x]	[ ]	Palaeontology and archaeology
[ ]	[x]	Animals and other organisms
[x]	[ ]	Clinical data
[x]	[ ]	Dual use research of concern

Methods

n/a	Involved in the study	
[x]	[ ]	ChIP-seq
[ ]	[x]	Flow cytometry
[x]	[ ]	MRI-based neuroimaging

Antibodies

Antibodies used

1. GAPDH, RRID: AB_10622025, Cell Signaling Technology, Catalog number: 5174, Clone ID: D16H11, Host organism: rabbit. Target antigen: human, mouse, rat, monkey. Applications: W, IHC-P, IF-IC.
2. eNOS, RRID: AB_2728756, Cell Signaling Technology, Catalog number: 32027, Clone ID: D9A5L, Host organism: rabbit. Target antigen: human, mouse, rat, bovine. Applications: W, IP, IF-IC.
3. p-eNOS (Ser1177), RRID: AB_823493, Cell Signaling Technology, Catalog number: 9570, Clone ID: C9C3, Host organism: rabbit. Target antigen: human, pig, bovine. Applications: W, IP, E-P.
4. AMPKα1, RRID: AB_722764, Abcam, Catalog number: ab32047. Host organism: rabbit. Target antigen: human, mouse, rat. Applications: IHC-P, IP, WB.
5. AMPKα2, RRID: AB_304055, Abcam, Catalog number: ab3760. Host organism: rabbit. Target antigen: human, mouse. Applications: ICC/IF, IHC-P, IP, WB.
6. p-AMPKα (Thr172), RRID: AB_2799368, Cell Signaling Technology, Catalog number: 50081, Clone ID: D4D6D, Host organism: rabbit. Target antigen: human, mouse, rat. Applications: W, IP, IHC-P.
7. caveolin-1, RRID: AB_2275453, Cell Signaling Technology, Catalog number: 3267, Clone ID: D46G3, Host organism: rabbit. Target antigen: human, mouse, rat. Applications: W, IP, IHC-P, IF-IC, F.
8. p16, RRID: AB_2799960, Cell Signaling Technology, Catalog number: 80772, Clone ID: D7C1M, Host organism: rabbit. Target antigen: human. Applications: W, IP, F.
9. p21, RRID: AB_823586, Cell Signaling Technology, Catalog number: 2947, Clone ID: 12D1, Host organism: rabbit. Target antigen: human. Applications: W, IP, IHC-P, IF-IC, F.
10. Ubiquitin, RRID: AB_11181462, Cell Signaling Technology, Catalog number: 3936, Clone ID: P4D1. Host organism: mouse. Target antigen: All. Applications: W, IHC-P.
11. DYKDDDDK Tag, RRID: AB_2572291, Cell Signaling Technology, Catalog number: 14793, Clone ID: D6W5B. Host organism: rabbit. Target antigen: All. Applications: W, IP, IHC-P, IF-IC, F, ChIP.
12. acetylated-lysine, RRID: AB_331805, Cell Signaling Technology, Catalog number: 9441. Host organism: rabbit. Target antigen: all. Applications: W, IP, IHC-P, IF-IC, ChIP, E-P.
13. HDAC1, RRID: AB_2756821, Cell Signaling Technology, Catalog number: 34589, Clone ID: D5C6U. Host organism: rabbit. Target antigen: human, mouse, rat. Applications: W, IP, IF-IC, ChIP, ChIP-seq.
14. p62, RRID: AB_2799160, Cell Signaling Technology, Catalog number: 39749, Clone ID: D1Q5S. Host organism: rabbit. Target antigen: human, mouse, rat. Applications: W, IP.
15. Sp1, RRID: AB_310773, Millipore, Catalog number: 07-645. Host organism: rabbit. Target antigen: human, mouse, rat. Applications: W, IP, IHC-P, IF-IC, F, ChIP.
16. Sp3, Abcam, Catalog number: ab227856. Host organism: rabbit. Target antigen: human. Applications: WB, IP, IHC-P, ICC/IF.
17. CD31, RRID: AB_726362, Abcam, Catalog number: ab28364. Host organism: rabbit. Target antigen: mouse, human, pig. Applications: IHC-Fr, IHC-P, ICC/IF, IHC-FoFr, WB.
18. p-ULK1 (Ser555), RRID: AB_10707365, Cell Signaling Technology, Catalog number: 5869, Clone ID: D1H4. Host organism: rabbit. Target antigen: human, mouse. Applications: W, IP.
19. ULK1, RRID: AB_11178668, Cell Signaling Technology, Catalog number: 8054, Clone ID: D8H5. Host organism: rabbit. Target antigen: human, mouse, rat. Applications: W, IP.
20. LC3A/B, RRID: AB_2728823, Cell Signaling Technology, Catalog number: 12741, Clone ID: D3U4C. Host organism: rabbit. Target antigen: human, mouse, rat. Applications: W, IHC-P, IF.
21. caspase 3, RRID: AB_2798429, Cell Signaling Technology, Catalog number: 14220, Clone ID: D3R6Y. Host organism: rabbit. Target antigen: human, mouse, rat. Applications: W, IP.
22. cleaved-caspase 3, RRID: AB_2341188, Cell Signaling Technology, Catalog number: 9661. Host organism: rabbit. Target antigen: human, mouse, rat. Applications: W, IP, IHC-P, IF-IC, F.
23. bradykinin B1 receptor, Santa Cruz Biotechnology Inc., Catalog number: sc-293196. Host organism: mouse. Target antigen: human, mouse, rat. Applications: W.
24. CD31, RRID: AB_307284, Abcam, Catalog number: ab9498. Host organism: mouse. Target antigen: human. Applications: Flow Cyt, ICC/IF, IHC-Fr, IHC-P.
25. AMPKα1/2, RRID: AB_1118940, Santa Cruz Biotechnology Inc., Catalog number: sc-74461. Host organism: mouse. Target antigen: human, mouse, rat. Applications: W, IP, IHC-P, IF-IC, F.
26. Sp1, RRID: AB_628271, Santa Cruz Biotechnology Inc., Catalog number: sc-420. Host organism: mouse. Target antigen: human, mouse, rat. Applications: W, IP, IHC-P, IF-IC, F.
27. Sp3, RRID: AB_628274, Santa Cruz Biotechnology Inc., Catalog number: sc-28305. Host organism: mouse. Target antigen: human, mouse, rat. Applications: W, IP, IHC-P, IF-IC, F.

Validation

1. GAPDH, RRID: AB_10622025, Cell Signaling Technology, Catalog number: 5174, Clone ID: D16H11, Host organism: rabbit. Target antigen: human, mouse, rat, monkey. Applications: W, IHC-P, IF-IC.
2. eNOS, RRID: AB_2728756, Cell Signaling Technology, Catalog number: 32027, Clone ID: D9A5L, Host organism: rabbit. Target antigen: human, mouse, rat, bovine. Applications: W, IP, IF-IC.
3. p-eNOS (Ser1177), RRID: AB_823493, Cell Signaling Technology, Catalog number: 9570, Clone ID: C9C3, Host organism: rabbit. Target antigen: human, pig, bovine. Applications: W, IP, E-P.
4. AMPKα1, RRID: AB_722764, Abcam, Catalog number: ab32047. Host organism: rabbit. Target antigen: human, mouse, rat. Applications: IHC-P, IP, WB.
5. AMPKα2, RRID: AB_304055, Abcam, Catalog number: ab3760. Host organism: rabbit. Target antigen: human, mouse. Applications: ICC/IF, IHC-P, IP, WB.
6. p-AMPKα (Thr172), RRID: AB_2799368, Cell Signaling Technology, Catalog number: 50081, Clone ID: D4D6D, Host organism: rabbit. Target antigen: human, mouse, rat. Applications: W, IP, IHC-P.
7. caveolin-1, RRID: AB_2275453, Cell Signaling Technology, Catalog number: 3267, Clone ID: D46G3, Host organism: rabbit. Target antigen: human, mouse, rat. Applications: W, IP, IHC-P, IF-IC, F.

8. p16, RRID: AB_2799960, Cell Signaling Technology, Catalog number: 80772, Clone ID: D7C1M, Host organism: rabbit. Target antigen: human. Applications: W, IP, F.
9. p21, RRID: AB_823586, Cell Signaling Technology, Catalog number: 2947, Clone ID: 12D1, Host organism: rabbit. Target antigen: human. Applications: W, IP, IHC-P, IF-IC, F.
10. Ubiquitin, RRID: AB_11181462, Cell Signaling Technology, Catalog number: 3936, Clone ID: P4D1. Host organism: mouse. Target antigen: All. Applications: W, IHC-P.
11. DYKDDDDK Tag, RRID: AB_2572291, Cell Signaling Technology, Catalog number: 14793, Clone ID: D6W5B. Host organism: rabbit. Target antigen: All. Applications: W, IP, IHC-P, IF-IC, F, ChIP.
12. acetylated-lysine, RRID: AB_331805, Cell Signaling Technology, Catalog number: 9441. Host organism: rabbit. Target antigen: all. Applications: W, IP, IHC-P, IF-IC, ChIP, E-P.
13. HDAC1, RRID: AB_2756821, Cell Signaling Technology, Catalog number: 34589, Clone ID: D5C6U. Host organism: rabbit. Target antigen: human, mouse, rat. Applications: W, IP, IF-IC, ChIP, ChIP-seq.
14. p62, RRID: AB_2799160, Cell Signaling Technology, Catalog number: 39749, Clone ID: D1Q5S. Host organism: rabbit. Target antigen: human, mouse, rat. Applications: W, IP.
15. Sp1, RRID: AB_310773, Millipore, Catalog number: 07-645. Host organism: rabbit. Target antigen: human, mouse, rat. Applications: W, IP, IHC-P, IF-IC, F, ChIP.
16. Sp3, Abcam, Catalog number: ab227856. Host organism: rabbit. Target antigen: human. Applications: WB, IP, IHC-P, ICC/IF.
17. CD31, RRID: AB_726362, Abcam, Catalog number: ab28364. Host organism: rabbit. Target antigen: mouse, human, pig. Applications: IHC-Fr, IHC-P, ICC/IF, IHC-FoFr, WB.
18. p-ULK1 (Ser555), RRID: AB_10707365, Cell Signaling Technology, Catalog number: 5869, Clone ID: D1H4. Host organism: rabbit. Target antigen: human, mouse. Applications: W, IP.
19. ULK1, RRID: AB_11178668, Cell Signaling Technology, Catalog number: 8054, Clone ID: D8H5. Host organism: rabbit. Target antigen: human, mouse, rat. Applications: W, IP.
20. LC3A/B, RRID: AB_2728823, Cell Signaling Technology, Catalog number: 12741, Clone ID: D3U4C. Host organism: rabbit. Target antigen: human, mouse, rat. Applications: W, IHC-P, IF.
21. caspase 3, RRID: AB_2798429, Cell Signaling Technology, Catalog number: 14220, Clone ID: D3R6Y. Host organism: rabbit. Target antigen: human, mouse, rat. Applications: W, IP.
22. cleaved-caspase 3, RRID: AB_2341188, Cell Signaling Technology, Catalog number: 9661. Host organism: rabbit. Target antigen: human, mouse, rat. Applications: W, IP, IHC-P, IF-IC, F.
23. bradykinin B1 receptor, Santa Cruz Biotechnology Inc., Catalog number: sc-293196. Host organism: mouse. Target antigen: human, mouse, rat. Applications: W.
24. CD31, RRID: AB_307284, Abcam, Catalog number: ab9498. Host organism: mouse. Target antigen: human. Applications: Flow Cyt, ICC/IF, IHC-Fr, IHC-P.
25. AMPKα1/2, RRID: AB_1118940, Santa Cruz Biotechnology Inc., Catalog number: sc-74461. Host organism: mouse. Target antigen: human, mouse, rat. Applications: W, IP, IHC-P, IF-IC, F.
26. Sp1, RRID: AB_628271, Santa Cruz Biotechnology Inc., Catalog number: sc-420. Host organism: mouse. Target antigen: human, mouse, rat. Applications: W, IP, IHC-P, IF-IC, F.
27. Sp3, RRID: AB_628274, Santa Cruz Biotechnology Inc., Catalog number: sc-28305. Host organism: mouse. Target antigen: human, mouse, rat. Applications: W, IP, IHC-P, IF-IC, F.

Eukaryotic cell lines

Policy information about cell lines and Sex and Gender in Research

Cell line source(s)	The HEK 293T/17 cell line (CRL-11268) was purchased from ATCC. The BAECs (HTX2256) was purchased from OTWO. HUVEC cells were isolated from normal human umbilical veins, which were collected from Qilu Hospital of Shandong University. Mouse lung endothelial cells and mouse retinal endothelial cells were isolated from indicated mouse.
Authentication	The cell lines were not authenticated.
Mycoplasma contamination	All cell lines tested negative for mycoplasma contamination.
Commonly misidentified lines (See ICLAC register)	No commonly misidentified cell lines are used in the study.

Animals and other research organisms

Policy information about studies involving animals; ARRIVE guidelines recommended for reporting animal research, and Sex and Gender in Research

Laboratory animals	The animals we used were all male mice of 6-8 weeks. The strains included VE-CAD-CreERT2+/Sp1(flox/flox)/Sp3(flox/flox), VE-CAD-CreERT2-/Sp1(flox/flox)/Sp3(flox/flox) and C57BL/6J. Mice were housed on a 12-h light/dark cycle and given ad libitum access to food and water. All animal protocols were approved by the Institutional Animal Care and Use Committee of Cheeloo College of Medicine, Shandong University.
Wild animals	The study did not involve wild animals.
Reporting on sex	The animals we used were all male mice according to similar studies in the field, so the findings in vivo applied to only male mice.
Field-collected samples	The study did not involve samples collected from the field.

Ethics oversight	Institutional Animal Care and Use Committee of Cheeloo College of Medicine, Shandong University.

Note that full information on the approval of the study protocol must also be provided in the manuscript.

Flow Cytometry

Plots

Confirm that:

- [x] The axis labels state the marker and fluorochrome used (e.g. CD4-FITC).
- [x] The axis scales are clearly visible. Include numbers along axes only for bottom left plot of group (a 'group' is an analysis of identical markers).
- [x] All plots are contour plots with outliers or pseudocolor plots.
- [x] A numerical value for number of cells or percentage (with statistics) is provided.

Methodology

Sample preparation	Cell apoptosis after AngII (MCE, Shanghai, China) stimuli was determined by using the FITC Annexin V Apoptosis Detection Kit I (BD Biosciences, CA, USA). Cells were harvested and resuspended in 100 μL of Annexin V binding buffer containing 5 μl propidium iodide (PI) and 5 μl Annexin V-FITC for 15 min at room temperature in the dark. An amount of 400 μl Annexin V binding buffer was added to each tube.
Instrument	Flow cytometry was performed and analyzed by using BD FACSCalibur.
Software	All data were analyzed by FlowJo (version V10).
Cell population abundance	At least 20,000 events were acquired for cells in the defined gate.
Gating strategy	Cellular debris were excluded by setting exclusion gates based on FSC and SSC. An untreated sample was run to adjust the voltage and gain for the FITC and PI detectors so that all cells can be detected in the bottom left quadrant. A treated sample stained with FITC alone was run to adjust the voltage and gain for the FITC detector so that the dead cells appear in the bottom right quadrant. A treated sample stained with PI alone was run to adjust the voltage and gain for the PI detector so that the dead cells appear in the top left quadrant. A treated sample stained with FITC and PI was run to adjust the compensation so that the live cells appear in the bottom left, the apoptotic cells appear in the bottom right, and the necrotic cells appear in the top right quadrants.

- [x] Tick this box to confirm that a figure exemplifying the gating strategy is provided in the Supplementary Information.
